# Screening a living biobank identifies cabazitaxel as a strategy to combat acquired taxol resistance in high-grade serous ovarian cancer

**DOI:** 10.1016/j.xcrm.2025.102160

**Published:** 2025-06-03

**Authors:** Anthony Tighe, Louisa Nelson, Robert D. Morgan, Bethany M. Barnes, I-Hsuan Lin, Samantha Littler, James Altringham, Jean Ling Tan, Joanne C. McGrail, Stephen S. Taylor

**Affiliations:** 1 Division of Cancer Sciences, School of Medical Sciences, Faculty of Biology, Medicine and Health, University of Manchester, Manchester Cancer Research Centre, 555 Wilmslow Road, Manchester M20 4GJ, UK; 2 Department of Medical Oncology, The Christie NHS Foundation Trust, Wilmslow Road, Manchester M20 4BX, UK; 3 Bioinformatics Core Facility, Faculty of Biology, Medicine and Health, University of Manchester, Michael Smith Building, Dover Street, Manchester M13 9PT, UK

**Keywords:** ovarian cancer, chromosome instability, drug resistance, centrosome amplification, spindle assembly checkpoint, intrinsic apoptosis pathway, *ABCB1*, MDR1, taxol, paclitaxel

## Abstract

The anti-mitotic agent taxol (paclitaxel) remains a cornerstone of ovarian cancer treatment. To tackle drug resistance and toxicity, second-generation targeted anti-mitotic agents and combination strategies are being explored but have yet to demonstrate meaningful clinical benefits. A limitation is the lack of a platform to compare strategies in models that capture disease heterogeneity. To overcome this, we screen 83 patient-derived *ex vivo* ovarian cancer models that exhibit extensive intra- and inter-patient heterogeneity, testing four distinct approaches to enhance taxol sensitivity. Inhibitors of the HSET kinesin or the Mps1 spindle assembly checkpoint kinase show minimal impact on the taxol sensitivity landscape. By contrast, Bcl-xL inhibition exerts a global anti-proliferative effect. Inhibition of the MDR1 drug efflux pump restores taxol sensitivity in models characterized by *ABCB1* overexpression. These MDR1-driven resistant models also respond to cabazitaxel, which is a poor MDR1 substrate, highlighting a potential therapeutic option for ovarian cancers with acquired taxol resistance.

## Highlights

• Taxol sensitivity landscape of 83 patient-derived ovarian cancer models defined
• HSET and Mps1 inhibitors have modest effects on the taxol sensitivity landscape
• A BH3 mimetic targeting Bcl-xL has a broad anti-clonogenic effect
• Models with *ABCB1*-mediated acquired taxol resistance are sensitive to cabazitaxel

## Graphical abstract

**Figure:**
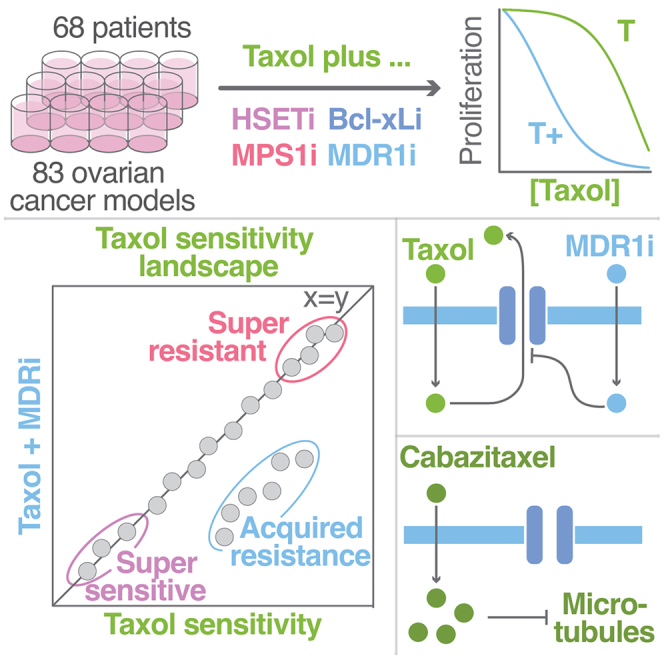

## Teaser

Tighe et al. screen a living biobank of 83 ovarian cancer models, systematically comparing four strategies to overcome intrinsic and/or acquired taxol resistance. Inhibiting drug efflux reverses acquired resistance in models overexpressing *ABCB1*. These models are also sensitive to cabazitaxel, providing an option for patients with acquired taxol resistance.

## Introduction

High-grade serous ovarian cancer (HGSOC) is the most prevalent and lethal ovarian cancer subtype.^1^ Standard treatment involves cytoreductive surgery followed by paclitaxel/carboplatin chemotherapy, with maintenance therapy where appropriate, e.g., PARP inhibitors for homologous recombination-deficient disease.^2^ While most tumors initially respond to chemotherapy, 10%–15% are intrinsically refractory, and many acquire resistance over time.^3^

Paclitaxel, originally known as taxol,^4^ is an anti-mitotic that stabilizes microtubules^5^ and is a cornerstone of ovarian cancer treatment, for both frontline therapy^6^^,^^7^ and the management of relapse.^8^^,^^9^ Despite initial efficacy, resistance is inevitable, leading to disease progression and poor outcomes. Overcoming taxol resistance, intrinsic or acquired, could provide significant clinical benefit. However, despite decades of research, strategies to enhance taxol efficacy are yet to be successfully implemented.^10^

The long-standing view is that taxol exerts anti-tumor effects by disrupting mitosis.^11^^,^^12^^,^^13^^,^^14^ Upon mitotic entry, microtubule dynamics increase, driving spindle assembly.^15^ Low-nanomolar taxol concentrations dampen microtubule dynamics, disrupt spindle assembly, and activate the spindle assembly checkpoint (SAC),^11^^,^^12^^,^^13^^,^^14^^,^^16^ leading to apoptosis, either during mitosis, after mitotic slippage, or following multipolar division.^17^ However, limited clinical efficacy of second-generation anti-mitotics prompted alternative hypotheses, including interphase or tumor microenvironment effects.^18^^,^^19^^,^^20^ Conversely, analyses of breast cancer biopsies collected during taxol treatment observed cell death following chromosome mis-segregation on multipolar spindles.^21^^,^^22^ Furthermore, acquired taxol resistance in ovarian cancer is associated with increased drug efflux activity,^23^^,^^24^ indicating a tumor-intrinsic mechanism.

Several targets have been explored to exploit mitosis, either independently of taxol or to enhance taxol efficacy.^25^ These include mitotic motors (e.g., Eg5/KSP, HSET/KIF1C, and CENP-E), mitotic kinases (e.g., Plk1, Aurora A, Aurora B, Mps1/TTK, and Nek2a), and E3 ligases (e.g., APC/C). Because taxol activates the intrinsic apoptosis pathway, targeting pro-survival factors (e.g., Mcl-1 and Bcl-xL) has also been explored.^26^ Additionally, taxol is a substrate for ABC transporters (e.g., MDR1/P-glycoprotein), and efflux inhibitors have been investigated as taxol sensitisers.^27^ Here, we compare four distinct strategies, targeting a mitotic motor, a mitotic kinase, a pro-survival factor, and a drug transporter.

Tumor cells frequently harbor supernumerary centrosomes, which are clustered to maintain bipolar spindle formation and chromosomal stability.^28^^,^^29^^,^^30^^,^^31^^,^^32^^,^^33^ HSET, a normally nonessential motor, becomes essential in cells with supernumerary centrosomes,^30^ making it an attractive target.^34^^,^^35^^,^^36^ HSET inhibitors can potentiate taxol efficacy *in vitro*^22^ but have not progressed to clinical trials. Given that extra centrosomes and multipolar mitoses are common HGSOC features,^37^^,^^38^^,^^39^^,^^40^ HSET inhibitors warrant further exploration.

HGSOC exhibits a high degree of chromosomal instability (CIN),^41^^,^^42^ an exploitable vulnerability^43^^,^^44^^,^^45^^,^^46^^,^^47^^,^^48^; aneuploid cancer cells are more dependent on SAC function,^49^ and inhibition of the SAC kinase Mps1 drives cells through aberrant mitosis,^50^ enhancing taxane sensitivity in various model systems.^45^^,^^51^^,^^52^^,^^53^^,^^54^^,^^55^^,^^56^^,^^57^^,^^58^^,^^59^ Notably, in an orthotopic triple-negative breast cancer model, Mps1 inhibition lowered the docetaxel dose required for tumor regression.^53^ While phase 1 clinical trials of Mps1 inhibitors have been completed in advanced malignancies,^60^^,^^61^ their therapeutic potential for HGSOC remains unexplored.

Taxol-induced cell death occurs via intrinsic apoptosis, which is modulated by oncogenes,^62^^,^^63^ including MYC, which is frequently amplified in HGSOC.^64^ MYC influences taxol sensitivity by downregulating Bcl-xL,^63^ a pro-survival factor often upregulated in taxol-resistant HGSOC.^65^^,^^66^^,^^67^ Targeting Bcl-xL is complicated by redundancy with other pro-survival proteins, particularly Mcl-1.^63^^,^^68^^,^^69^^,^^70^ However, because Mcl-1 is degraded during a protracted mitosis,^71^^,^^72^^,^^73^^,^^74^^,^^75^^,^^76^^,^^77^ post-mitotic survival becomes particularly reliant on Bcl-xL.^63^^,^^70^ Since taxol delays mitosis, Bcl-xL inhibition may enhance its efficacy. Indeed, genetic screens identified Bcl-xL as a determinant of taxol sensitivity,^67^ and pharmacological inhibitors enhance taxol-induced apoptosis in multiple models.^66^^,^^70^^,^^73^^,^^78^^,^^79^^,^^80^^,^^81^ Moreover, as HGSOC cells undergo prolonged mitosis even in the absence of taxol,^37^ Bcl-xL inhibitors may have monotherapy efficacy.^82^ While Bcl-xL inhibitors have entered clinical trials,^83^^,^^84^ the subset of HGSOC most likely to benefit remains unclear.

HGSOC drug resistance is often associated with *ABCB1* translocations, leading to MDR1/P-glycoprotein overexpression.^23^^,^^24^ While MDR1 inhibition can reverse efflux-mediated taxol resistance in preclinical models,^24^^,^^67^^,^^85^^,^^86^^,^^87^ clinical trials were unsuccessful.^27^^,^^88^ However, these studies did not select patients based on *ABCB1* overexpression.^88^ With the advent of second- and third-generation MDR1 inhibitors, and an appreciation of the need for predictive biomarkers, revisiting this strategy is warranted—particularly given that *ABCB1* is overexpressed in ∼20% of relapsed HGSOC.^24^

Despite extensive research, these therapeutic strategies are yet to translate into clinical benefit. A major challenge has been the lack of a systematic approach to compare and prioritize strategies using preclinical models that capture disease heterogeneity. To address this, we established a living biobank of ovarian cancer models (OCMs)—patient-derived *ex vivo* cultures with extensive proliferative potential that retain key HGSOC features.^37^^,^^89^ OCMs enable functional and multi-omics analyses and are clinically annotated, allowing integration of drug sensitivity, molecular features, and clinical data. Here, we screen a diverse panel of OCMs to evaluate inhibitors targeting HSET, Mps1, Bcl-xL, and MDR1.

## Results

### Analyzing the taxol sensitivity landscape of ovarian cancer

**Figure 1:**
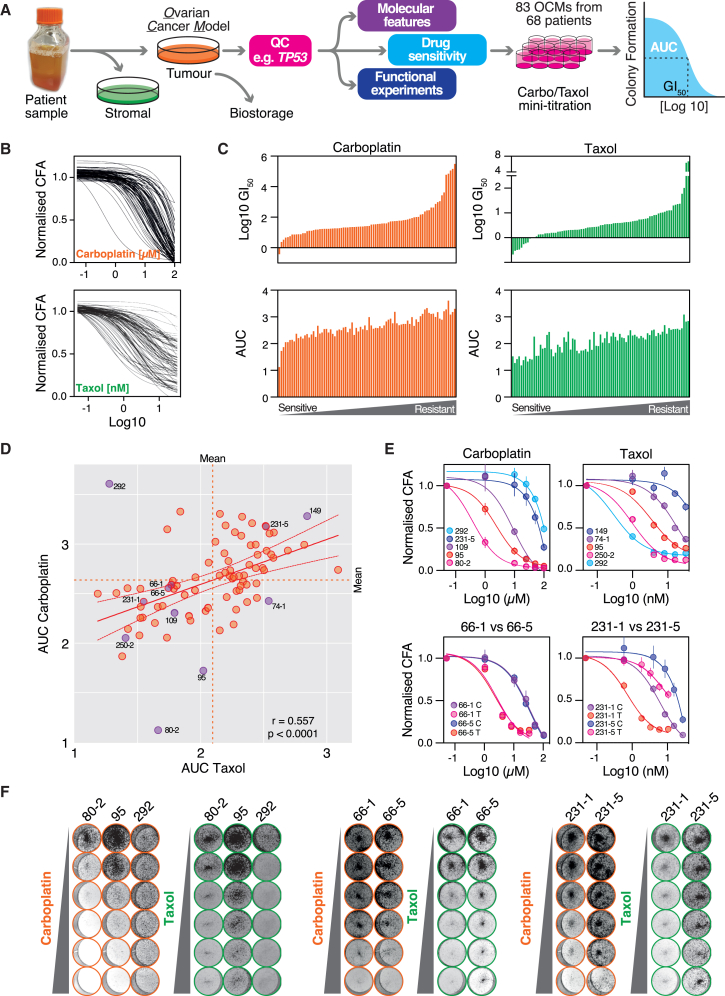
Analyzing the taxol sensitivity landscape of ovarian cancer (A) Workflow for sample processing, OCM validation, and drug sensitivity screen. (B) Carboplatin and taxol dose-response curves for 83 OCMs. (C) Bar charts showing GI_50_ (ranked ordered) and AUC values for carboplatin and taxol. (D) *xy* plot showing mean AUC values for carboplatin and taxol, with linear regression, 95% confidence bands, and Spearman r correlation. *p* < 0.0001. (E) Dose-response curves for carboplatin (C) and taxol (T) for selected OCMs, showing mean and standard error. (F) Exemplar CFA images for selected OCMs following a 6-day exposure to carboplatin or taxol. https://creativecommons.org/licenses/by/4.0/ (A) adapted from Nelson et al. 2020 (license at https://creativecommons.org/licenses/by/4.0/). Values throughout derived from at least three biological replicates. See Figures S1 and S2; Table S1.

To explore the taxol sensitivity landscape across a diverse range of ovarian cancers, we developed a pipeline to collect fresh biopsies from patients undergoing treatment at The Christie hospital and a workflow to generate proliferative tumor fractions (Figure 1A). Here, we focused on 83 OCMs from 68 patients. Most OCMs were generated from HGSOC, while 12 were derived from other subtypes (Table S1). Most OCMs were generated from ascites, but four were from solid samples, and while 14 OCMs are chemo-naive, most were acquired from pre-treated patients (Table S1; Figure S1). The OCMs were exposed to taxol and carboplatin titrations (Figure 1A), analyzed via colony formation assay (CFA) generating dose-response curves (Figure 1B) from which GI_50_ and area under the curve (AUC) values were calculated (Figure 1C), metrics that were very strongly correlated (Figure S2A). The median taxol GI_50_ was 3.20 nM, with a range of 0.21–102 nM (Figure S2B). Across the panel, there was a moderate correlation between carboplatin and taxol sensitivity (Figure 1D). Crosschecking the scatterplot with the dose-response curves and assay plates (Figures 1E and 1F) confirmed that it reflects the underlying data, thereby visualizing the taxol/carboplatin sensitivity landscape of ovarian cancer.

### OCM *ex vivo* drug sensitivity profiling captures inter-patient and intra-tumor heterogeneity

The taxol sensitivity range across the OCMs is approximately 500-fold (Figure S2B). Does this range reflect *in vivo* tumor sensitivity? While 55 patients are represented by a single OCM with no available comparators, 28 OCMs are derived from 13 patients (Table S1; Figure S1 and see STAR methods), providing an opportunity to compare models from the same patient. We predicted that if *ex vivo* profiling reflects the sampled tumors, then OCMs derived from the same patient should behave similarly, especially if there was little intervening time and/or chemotherapy.

Inspection of the *xy* plot revealed that for 9 of the 13 subsets, each OCM was associated with its respective partner (Figure S2C, left panel). For example, the pairs from 118 and 124, which were derived from pre- and post-treatment low-grade serous ovarian cancer (LGSOC), were closely aligned. Regarding carboplatin, these OCMs are among the most resistant, consistent with the clinical data indicating that LGSOCs are intrinsically resistant to traditional chemotherapy.^90^^,^^91^ Two OCMs generated in parallel from spatially resolved solid samples, 361a and 361b, were also tightly associated (Figure S2C, left panel).

Interestingly, for four subsets (64, 74, 231, and 341), the matched samples occupied different positions on the plot (Figure S2C, right panel). 231-1 and 231-5 were generated from chemo-naive and post-relapse biopsies collected 18 months apart (Figure S1). These OCMs have different dose-response curves and CFA images (Figures 1E and 1F), with the post-relapse OCM.231-5 ∼18-fold more resistant than chemo-naive OCM.231-1. They are morphologically distinct, consistent with *in vivo* expansion of a more resistant subclone. 341-1 and 341-3 were also derived from chemo-naive and post-treatment samples, again consistent with the expansion of a more resistant subclone following chemotherapy. The two triplet subsets also make interesting exceptions. While 64-1 was relatively resistant, despite the differences exhibited by 64-3-Ep+ and 64-3-Ep− (STAR Methods), they displayed almost identical drug sensitivity. In addition, while OCMs 74-1 and 74-5 are morphologically similar and relatively resistant to taxol, OCM.74-3 is morphologically distinct and relatively taxol sensitive (Figure S2C), possibly reflecting expansion of a different subclone.

Because paired models from 9 of the 13 subsets behave similarly, the OCM generation workflow and the *ex vivo* analysis do generate models and drug sensitivity profiles reflective of the *in vivo* tumors. Moreover, when comparing longitudinal OCMs from the same patient, if taxol sensitivity differs, this likely reflects intra-tumor heterogeneity. In turn, when comparing OCMs from different patients, different taxol sensitivities demonstrate inter-patient heterogeneity.

### Global analysis of taxol modulation strategies

**Figure 2:**
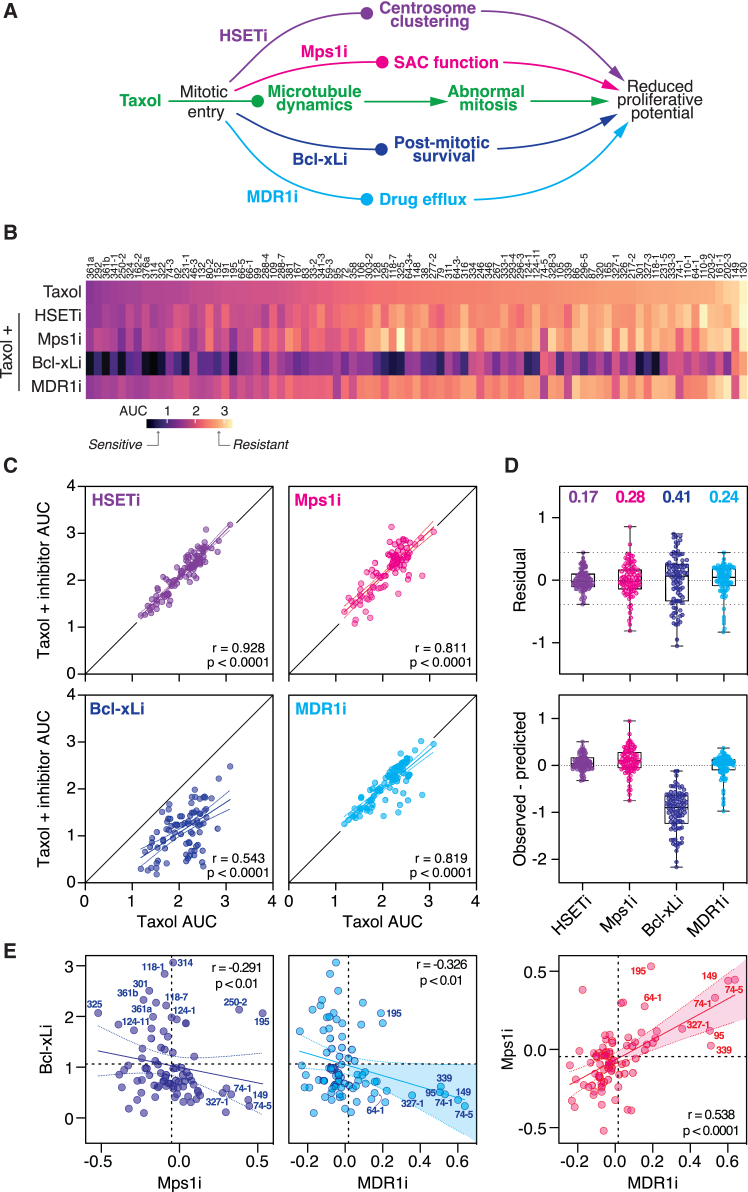
Global analysis of taxol modulation strategies (A) Mechanism of action of taxol and inhibitors targeting HSET, Mps1, Bcl-xL, and MDR1. (B) Heatmap of AUC values for OCMs treated with taxol plus HSETi, Mps1i, Bcl-xLi, or MDR1i, ranked by taxol sensitivity. (C) *xy* plots showing sensitivity to taxol versus taxol + inhibitor. Solid black lines show *x = y*, solid-colored lines show linear regression, r represents Pearson correlation. *p* < 0.0001 (all panels). (D) Box-and-whisker plots with interquartile ranges showing residuals based on the linear regression model (upper graph; values represent standard deviation as a measure of variance), and the *y-x* difference (lower graph). (E) *xy* plots showing log2-transformed AUC ratios for Bcl-xLi/Mps1i, Bcl-xLi/MDR1i, and Mps1i/MDR1i. Spearman r is used to measure correlation. Values derived from three biological replicates; *p* < 0.01 (left and center) and *p* < 0.0001 (right). See Figures S3, S4, and S5.

To explore whether the taxol sensitivity landscape can be modulated, we tested four combination strategies, adding an additional agent at a single concentration to the taxol titrations (STAR Methods). We focused on inhibitors targeting (1) HSET (AZ82; hereafter HSETi^92^), (2) Mps1 (AZ3146; Mps1i^93^), (3) Bcl-xL (A-1155463; Bcl-xLi^94^), and (4) MDR1 (elacridar; MDR1i^95^) (Figure 2A). To visualize the effects, we plotted AUC values as a heatmap (Figure 2B) and log2 transformed the AUC ratios of taxol alone versus the combination, rank ordered by the extent of sensitization (Figure S3). While the HSET, Mps1, and MDR1 inhibitors had both sensitization and desensitization effects, albeit with different magnitudes, there was global sensitization to the Bcl-xLi. To explore further, we generated *xy* graphs, plotting the AUC for the combination (the *observed* value) against the AUC for taxol alone (the *predicted* value) (Figure 2C). From these, we calculated correlation coefficients and generated linear regression models. We then calculated (1) the residual, i.e., the difference between the observed value and the value predicted by the linear regression model, and (2) the difference between observed and predicted value, i.e., *y*−*x* (Figure 2D).

For the HSETi, the correlation between taxol and taxol plus HSETi was very strong, with all the values close to *x* = *y* (Figure 2C). The residuals were small and displayed low variability (Figure 2D), suggesting that the HSETi had little impact on the taxol sensitivity landscape. To confirm target engagement, we analyzed BT549 cells and a subset of OCMs by immunofluorescence (Figure S4A). In controls, we observed bipolar spindles with multiple centrosomes, consistent with HSET-dependent clustering. In inhibitor-treated cells, we observed de-clustered centrosomes and multipolar spindles. Despite this, inspection of dose-response curves and CFA images confirmed that the HSETi had little impact on taxol sensitivity, in OCMs that were relatively resistant or sensitive to taxol alone (Figures S4B and S4C). This was unexpected but may reflect using the HSETi at a single concentration. Therefore, we tested a range of concentrations in eight OCMs (Figure S4D). Higher concentrations were toxic in the absence of taxol, possibly reflecting off-target effects. At intermediate concentrations, we saw evidence of sensitization only in OCM.149 (Figure S4D). Synergy analysis confirmed this; averaging synergy scores from Loewe, Bliss, highest single agent (HSA), and zero interaction potency (ZIP) models yielded a mean of 11.8 for OCM.149 but values less than zero for the other OCMs (Figure S5). Thus, while HSET inhibition has little impact on the overall OCM taxol sensitivity landscape, we cannot rule out the possibility that a small subset of HGSOC could be taxol sensitized. Exploring this further will benefit from more potent HSET inhibitors.

For Mps1i, the correlation between taxol and taxol plus Mps1i was also very strong (Figure 2C). However, several OCMs were displaced from the *x* = *y* line, and the residuals displayed more variability, with 10 extending beyond the range observed with the HSETi (Figure 2D). Interestingly, while 8 OCMs had negative residuals, indicating sensitization, two had positive values, indicating desensitization. We explore this further in the following section. For the Bcl-xLi, the correlation between taxol and taxol plus Bcl-xLi was moderate; strikingly however, all the values fell below *x* = *y* (Figure 2C). The linear regression residuals showed extensive variation, and the *y*-*x* residuals were all less than zero (Figure 2D). These observations reflect the heatmap (Figure 2B), indicating that Bcl-xLi has a global sensitization effect. We also explore this further below. The correlation between taxol and taxol plus MDR1i was very strong but with several OCMs displaced. The variation exhibited by the residuals was similar to that observed with the Mps1i, but skewed toward negative values, i.e., sensitization. We explore this further in the following section.

To explore potential relationships between the Mps1i, Bcl-xLi, and MDR1i effects, we plotted the log2-transformed AUC ratios calculated earlier (Figure S3) as *xy* scatter graphs for each of the pairwise combinations (Figure 2E). Interestingly, there was a moderate positive correlation for Mps1i/MDR1i; we revisit this in the discussion. Meanwhile, we conclude that the OCM taxol sensitivity landscape can be modulated; while the HSETi had little effect, the Mps1, Bcl-xL, and MDR1 inhibitors yielded substantial changes, with Bcl-xLi having a global sensitization effect. By contrast, Mps1i and MDR1i had more selective effects, modulating a small subset of OCMs.

### Mps1 inhibition sensitizes a subset of OCMs to taxol

**Figure 3:**
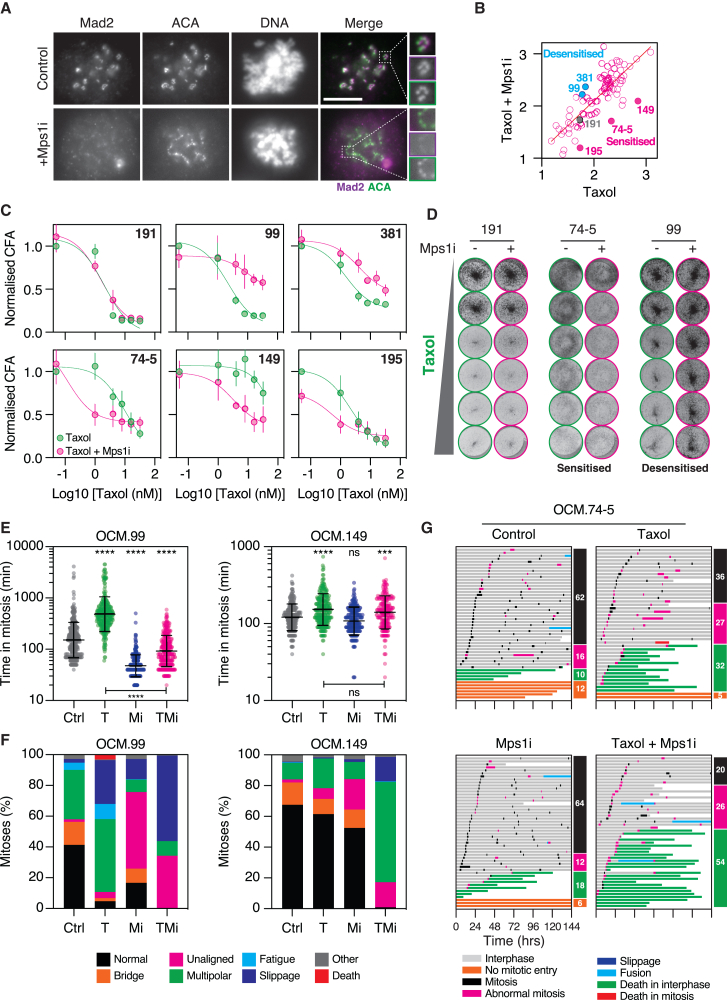
Mps1 inhibition sensitizes a subset of OCMs to taxol (A) Mitotic OCM.74-1 cells treated ± Mps1i showing Mad2 and centromeres (ACA). Insets show higher magnification of kinetochores. Bar 10 μm (B) *xy* graph plotting AUC for taxol versus taxol plus Mps1i, as shown in Figure 2C, highlighting OCMs selected for further analysis. (C) Dose-response curves for highlighted OCMs. Mean and SD from three biological replicates. (D) Exemplar CFA of selected OCMs after 6-day exposure to taxol ± Mps1i. (E) Time-lapse analysis of GFP-H2B-expressing OCM.99 and OCM.149 either untreated (Ctrl) or exposed to taxol (T, 4 nM), Mps1i (Mi, 2 μM), or the combination (TMi). Time in mitosis was measured from nuclear envelope breakdown (NEBD) to anaphase onset, ≥200 cells analyzed per condition. Lines represent median and interquartile range. One-way ANOVA, n.s. *p* > 0.05, ∗∗∗*p* < 0.001, ∗∗∗∗*p* < 0.0001. (F) Bar graph quantifying mitotic abnormalities. (G) Cell fate profiling of OCM.74-5, either control or treated with taxol (1 nM), Mps1i (2 μM), or the combination. Horizontal bars represent a single cell (50 cells per condition), with colors indicating cell behavior. Numbers in colored boxes show the percentage of cells with indicated behavior. Data in (A), (E), (F), and (G) derived from one biological replicate. See Figure S6.

To understand the Mps1i effect, we first ensured that AZ3146 was eliciting the anticipated on-target effect; indeed, in Mps1i-treated OCMs, recruitment of O-Mad2 to kinetochores was diminished (Figures 3A and S6).^93^ To confirm the Mps1i effects, we focused on six OCMs (Figure 3B). Importantly, interrogation of dose-response curves and CFA images confirmed that OCMs 74-5, 149, and 195 were sensitized to taxol by the Mps1i, while OCMs 99 and 381 were desensitized, and 191 was unaffected (Figures 3C and 3D).

Time-lapse analysis showed that for desensitized OCM.99, the average time in mitosis was 230 min, and exposure to 4 nM taxol delayed this to 685 min (Figure 3E). Consistent with SAC override, the Mps1i accelerated mitosis, in the absence and presence of taxol. Notably, the Mps1i also changed the phenotype of taxol-treated cells, reducing multipolar segregations from 47% to 10% (Figure 3F). Analysis of sensitized OCM.149 showed that taxol also delayed mitotic timing, and this was accelerated by the Mps1i (Figure 3E), although the effect was less profound. The Mps1i also changed the phenotype of taxol-treated cells, but the number of multipolar segregations increased from 19% to 65% (Figure 3F).

In breast cancer cells, taxol sensitivity correlates with multipolar mitoses.^21^^,^^22^^,^^96^ Here, we observe a similar phenomenon: in the desensitized OCM, the Mps1i reduced multipolar segregations, while in the sensitized case, multipolar segregations increased. This is not correlated with mitotic timing; while the sensitized OCM showed only modest timing differences, the Mps1i markedly accelerated mitosis in the desensitized case (Figure 3E). One possibility is that excessive acceleration provides insufficient time for spindle assembly, and, indeed, slippage became the dominant phenotype in the taxol/Mps1i-treated OCM.99 (Figure 3F). Because slippage leads to tetraploidization and whole-genome doubling, perhaps this is more likely to yield viable progeny than the highly unequal segregation events mediated by a multipolar spindle. Indeed, cell fate profiling of sensitized OCM.74-5 showed that the taxol/Mps1i combination increased the frequency of apoptosis, which occurred after abnormal divisions rather than slippage (Figure 3G).

Thus, inhibiting Mps1 sensitizes a small subset of HGSOC models to taxol, with a plausible mechanism being an increase in multipolar mitoses leading to apoptosis. However, developing this into a therapeutic strategy is complicated by the observation that Mps1 inhibition can also desensitize some OCMs to taxol.

### Bcl-xL inhibition has a broad-ranging sensitization effect

**Figure 4:**
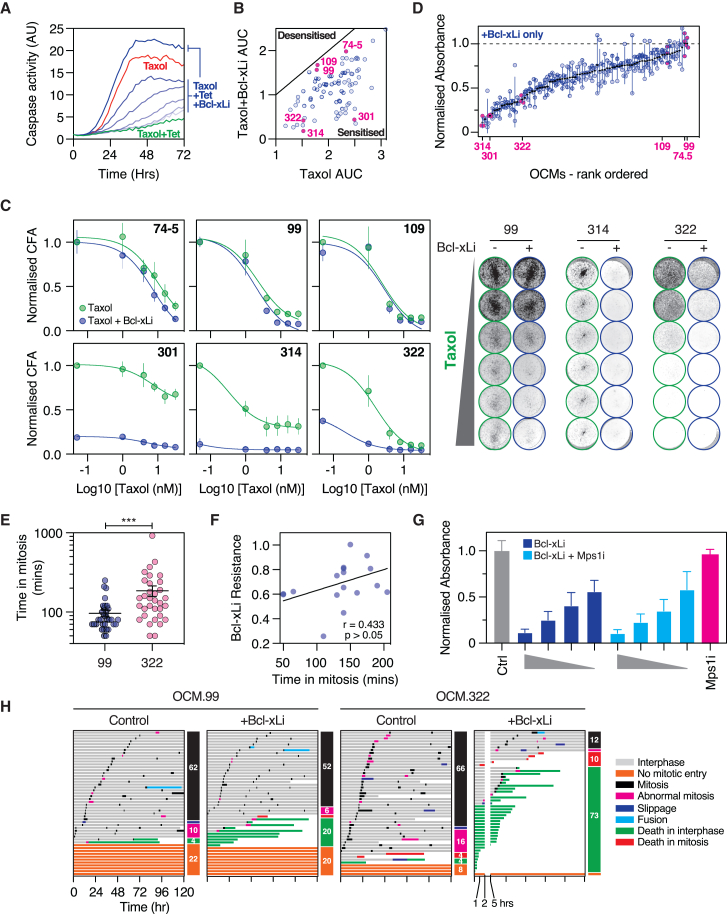
Bcl-xL inhibition has a broad-ranging sensitization effect (A) Graph showing apoptosis in RKO cells expressing tetracycline (Tet)-inducible Bcl-xL following exposure to taxol, taxol + Tet, and taxol + Tet + Bcl-xLi. (B) *xy* graph plotting AUC for taxol against taxol plus Bcl-xLi, as shown in Figure 2C, highlighting OCMs selected for further analysis. (C) Left: dose-response curves for highlighted OCMs. Data are mean and SD from three biological replicates. Right: exemplar CFA images after 6-day exposure to taxol ± Bcl-xLi. (D) Graph plotting effect of 100 nM Bcl-xLi alone with OCMs ranked by sensitivity, showing mean and SD from three biological replicates. (E) Graph showing duration of the first mitosis for OCMs 99 and 322. Each symbol represents an individual cell, with >30 cells analyzed. The bars show mean and SEM from one experiment. Paired t test. ∗∗∗*p* < 0.001. (F) *xy* graph plotting the response (mean normalized absorbance) of 17 OCMs to 100 nM Bcl-xLi against median time spent in mitosis. Pearson r is used to measure the correlation. *p* > 0.05. (G) Bar graph quantitating clonogenic potential of OCM.322 exposed to Bcl-xLi (10, 25, 50, and 100 nM) ± Mps1i (2 μM). Bars show mean and SD from three biological replicates. (H) Cell fate profiling of OCMs 99 and 322, untreated or treated with 100 nM Bcl-xLi for 5 days. Horizontal bars represent a single cell (50 cells per condition), with colors indicating cell behavior. Numbers in colored boxes show the percentage of cells with the indicated behavior. Data in (A), (E), (F), and (H) show one biological replicate. See Figures S5 and S7.

To characterize the Bcl-xLi effect, we first verified that A-1155463, a BH3 mimetic that targets Bcl-xL but not Bcl2 or Mcl-1,^94^ suppresses Bcl-xL activity at the concentrations deployed. Importantly, while tetracycline-mediated overexpression of a Bcl-xL cDNA suppressed taxol-induced apoptosis in RKO cells, this protective effect was reversed in a dose-dependent manner by the Bcl-xLi, consistent with on-target activity (Figure 4A). To confirm the Bcl-xLi effect observed in the screen, we focused on six OCMs (Figure 4B). Interrogation of dose-response curves and CFA images confirmed that OCMs 301, 314, and 322 were sensitized to taxol, while 74-5, 99, and 109 were only marginally sensitized (Figure 4C), consistent with the latter OCMs’ proximity to the *x* = *y* line (Figure 4B).

We noted however that for the three sensitized models, in the zero-taxol conditions, Bcl-xLi alone exerted a substantial anti-clonogenic effect (Figure 4C). Therefore, we extracted all the zero-taxol values from the screen, revealing that 100 nM of the Bcl-xLi had an inhibitory effect in most cases (Figure 4D). This was surprising, as in prior studies, an exogenous pro-apoptotic stimulus was required for Bcl-xL inhibitors to have a potentiating effect.^70^^,^^97^ Interestingly, we previously showed that, compared with established cell lines, OCMs spend a protracted amount of time in mitosis.^37^ We reasoned therefore that perhaps these inherent delays were sufficient to degrade Mcl-1 and create a Bcl-xL dependency without the need for a taxol-induced delay. Consistent with this, hypersensitive OCM.322 cells typically spent longer in mitosis than insensitive OCM.99 cells (Figure 4E). However, mitotic timings measured here and in our previous study^37^ did not significantly correlate with Bcl-xLi resistance (Figure 4F). In addition, exposing OCM.322 to Mps1i to accelerate mitosis did not reduce the Bcl-xLi-induced anti-proliferative effect (Figure 4G). Thus, OCM sensitivity to Bcl-xLi is not explained by protracted mitoses. Nevertheless, cell fate profiling showed that exposing OCM.322 to the Bcl-xLi alone increased apoptosis from 8% to 83% (Figure 4H). Because 100 nM Bcl-xLi alone had a penetrant effect in many cases, we analyzed a subset of eight OCMs at 10, 50, and 100 nM and five OCMs at 1, 5, 10, 25, 50, and 100 nM Bcl-xLi (Figure S7). While the Bcl-xLi effect was titratable in some cases, e.g., OCM.322 (Figure S7A), we still did not see a substantial taxol sensitization effect. Synergy analysis confirmed this (Figures S5A–S5C). Thus, the apparent taxol sensitization effect uncovered by the screen is largely driven by the ability of Bcl-xLi monotherapy to directly induce cell death, without an additional exogenous pro-apoptotic stimulus. Indeed, some OCMs (e.g., 327-3) were sensitive to low-nanomolar Bcl-xLi in the absence of taxol (Figure S5D).

### Inhibition of MDR1i-mediated drug efflux re-sensitizes a subset of OCMs to taxol

**Figure 5:**
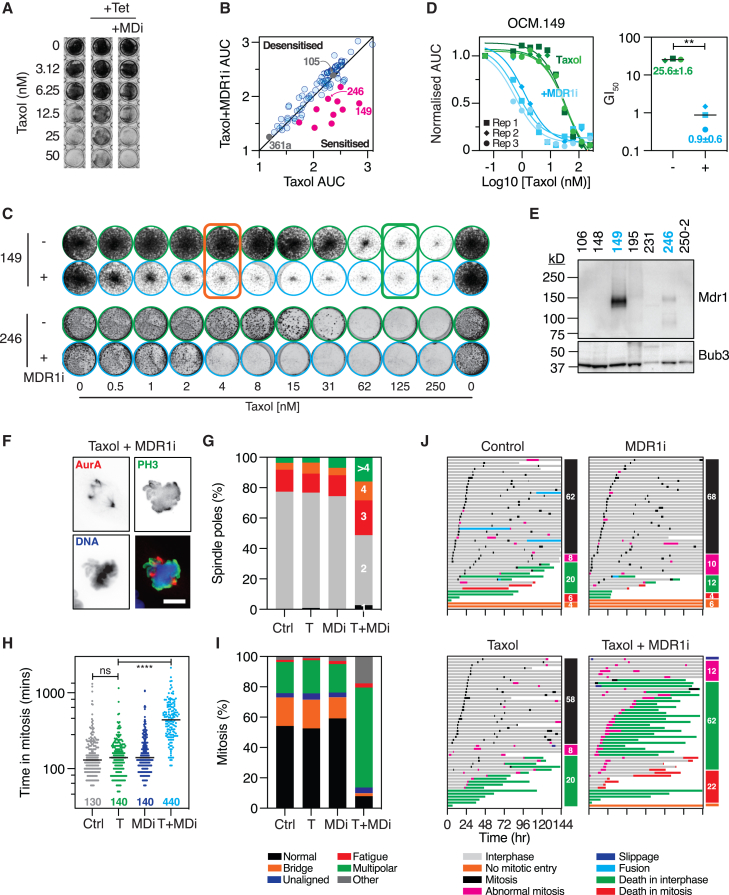
Inhibition of MDR1i-mediated drug efflux re-sensitizes a subset of OCMs to taxol (A) CFA of RKO cells expressing tetracycline-inducible MDR1 exposed to a taxol titration ±1 μg/mL tetracycline (Tet) and 250 nM MDR1i (MDi, elacridar). (B) *xy* graph plotting AUC for taxol against taxol plus MDR1i, as shown in Figure 2C, highlighting selected OCMs. (C) CFA of OCMs 149 and 246 exposed to taxol ±250 nM MDR1i. Red/green boxes highlight doses referred to in the text. (D) Taxol dose-response curves for OCM.149 ± MDR1i (left), with proliferation measured by time-lapse imaging of cells expressing GFP-Histone H2B (GFP-H2B), imaging every 4 h for 136 h. Data are from three biological replicates. Mean GI_50_ values for OCM.149 (right). Paired t test ∗∗*p* < 0.01. (E) Immunoblot of MDR1 in selected OCMs. Bub3 is a loading control. (F) Mitotic OCM.149 cell stained for Aurora A and Phospho-Histone H3 Ser10 following treatment with 4 nM taxol and MDR1i. Bar 10 μm. (G) Bar graph quantifying the number of spindle poles in OCM.149 following exposure to 4 nM taxol (T), MDR1i (MDi), or the combination (T + MDi). At least 100 cells were analyzed per condition. (H) Time-lapse analysis of OCM.149 expressing GFP-H2B either untreated (Ctrl) or exposed to taxol (T, 4 nM), MDR1i (MDi), or the combination (T + MDi). Time in mitosis was measured from nuclear envelope breakdown (NEBD) to anaphase onset, with the median time indicated. At least 141 cells analyzed per condition. One-way ANOVA, n.s. *p* > 0.05, ∗∗∗∗*p* < 0.0001. (I) Bar graph quantifying mitotic abnormalities. (J) Cell fate profiling of OCM.149 either untreated (control) or treated with taxol (4 nM), MDR1i, or the combination for 6 days. Horizontal bars represent a single cell (50 cells per condition), with colors indicating cell behavior. Numbers in colored boxes show the percentage of cells with the indicated behavior. Data in (A) and (E)–(J) derived from one biological replicate. See Figures S5, S7, and S8.

To characterize the MDR1i effect, we first verified that elacridar was inhibiting drug efflux at the concentrations deployed. While tetracycline-mediated overexpression of *ABCB1* suppressed taxol-induced toxicity in RKO cells, this was reversed by 250 nM MDR1i (Figures 5A, S8A, and S8B). Overexpression of *ABCB1* did not suppress cisplatin-induced toxicity (Figure S8C), consistent with the substrate profile of MDR1.^27^^,^^98^ Based on the taxol/Mdr1i screen, we selected several OCMs for further analysis (Figure 5B). Interrogation of the titrations confirmed that OCMs 149 and 246 were sensitized by MDR1i, while 105 and 361a were not (Figure S8d), with 10-point dose-response assays confirming the ability of the MDR1i to resensitize OCMs 149 and 246 to low-nanomolar concentrations of taxol (Figures 5C and 5D).

Next, we asked whether the MDR1i effect was consistent with inhibition of MDR1. First, while the screen was performed at 250 nM elacridar, MDR1i-induced taxol toxicity was dose dependent, manifesting at 8–15 nM (Figure S8E). Indeed, synergy analysis of OCM.149 yielded an average value of 37.1, indicating strong synergy between taxol and MDR1i (Figures S5B and S7B). Second, following exposure to taxol and MDR1i, we observed multi-nucleated cells and nuclear atypia (Figure S8F), consistent with the disruption of microtubule-dependent processes. Finally, while elacridar can inhibit several *ABC*-family member transporters,^27^ both 149 and 246 overexpress MDR1 (Figures 5E and S8G), consistent with the upregulation of *ABCB1* driving the taxol resistance. Therefore, the MDR1i-dependent taxol sensitization is via inhibition of MDR1, demonstrating that inhibition of drug efflux mechanisms can re-sensitize a subset of OCMs to taxol.

To further explore the phenotypic consequences of the taxol/MDR1i combination, we analyzed OCM.149 in more detail, reasoning this would expose the mechanism by which taxol exerts its anti-proliferative effect in HGSOC. There has been considerable speculation that the anti-tumor effects of taxol are not due to disruption of tumor cell mitoses but rather via the tumor microenvironment and/or non-mitotic effects.^18^^,^^19^^,^^20^ If taxol sensitivity was via mitotic defects, then these should manifest at 4 nM taxol plus MDR1i but not at 4 nM taxol alone (Figure 5C, red box). Immunofluorescence revealed that in OCM.149, most mitotic cells built bipolar spindles, and this was largely unaffected by taxol or MDR1i alone. The combination of 4 nM taxol plus MDR1i however resulted in a marked increase in multipolar spindles (Figures 5F and 5G). Time-lapse microscopy revealed that the combination induced a substantial mitotic delay and a marked increase in multipolar divisions (Figures 5H and 5I). Finally, cell fate profiling revealed that the taxol/MDR1i combination markedly increased apoptosis, with 22% of cells dying in mitosis and 62% dying after an abnormal cell division (Figure 5J). Interestingly, at 125 nM taxol, where the MDR1i had little effect (Figure 5C green box), presumably due to saturation of the drug efflux mechanism, cell fate profiles plus/minus MDR1i were more similar, with longer mitotic delays, mitotic slippage, and apoptosis (Figure S8H). Nevertheless, based on the differential behavior of cells at the lower taxol concentration, we conclude that taxol toxicity in HGSOC is driven by a tumor-cell-intrinsic phenomenon, characterized by mitotic delays followed by multipolar division and post-mitotic apoptosis.

### Acquired taxol resistance correlates with *ABCB1* overexpression and accumulated taxol exposure

**Figure 6:**
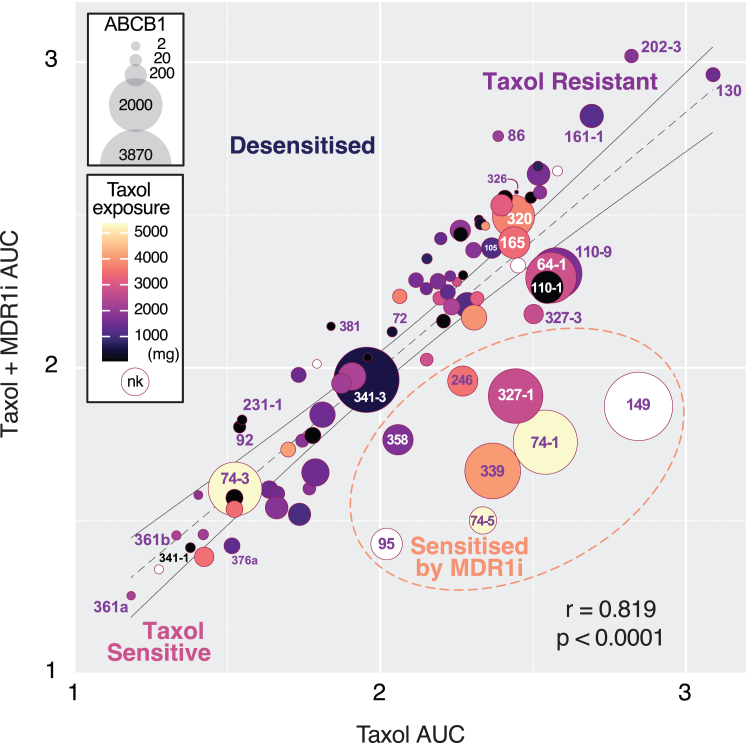
Acquired taxol resistance correlates with *ABCB1* overexpression and accumulated taxol exposure Multi-variable graph plotting AUC for taxol on the *x* axis against AUC for taxol + MDR1i on the *y* axis. Bubble size represents *ABCB1* expression levels (normalized read count from RNA sequencing), and color represents patient exposure to taxol (mg) prior to sample collection. For six OCMs, taxol exposure was not known (nk). OCMs in the bottom right quadrant are sensitized to taxol by the MDR1i. AUC values derived from at least three biological replicates. Lines show linear regression with 95% confidence intervals and r represents Pearson correlation; *p* < 0.0001. See Figure S9; Table S2.

To explore the drug efflux re-sensitization phenomenon more broadly, we integrated three metrics, namely (1) *ex vivo* re-sensitization, determined by the taxol/MDR1i residual, (2) *ABCB1* expression levels, and (3) the accumulated milligrams of taxol each patient was exposed to prior to collection of the biopsy that yielded the respective OCM (Figures 6, S9A, and S9B). We categorized nine OCMs as “super expressers” with read counts >1,000 and a further 11 as “high expressers” with read counts >300 (Figure S9B). Based on residuals greater than one standard deviation from the mean, we identified the “top 10” MDR1i responders (Figure S9B). In terms of taxol exposure, 19 patients fell into the top quartile, with accumulated taxol exposures greater than 2,800 mg (Figures S9A and S9B). Plotting these three metrics as pairwise *xy* graphs revealed three significant correlations: moderate between *ABCB1* expression and re-sensitization; and weak between *ABCB1* expression and taxol exposure and between re-sensitization and taxol exposure (Figure S9C).

In a multivariable plot of AUC for taxol/MDR1i against the AUC for taxol alone, with *ABCB1* expression represented by bubble size and taxol exposure represented by color (Figure 6), most OCMs lay close to the linear regression line and were represented by relatively smaller, darker colored symbols. Strikingly, the OCMs sensitized by MDR1i, which manifest in the lower right sector, were represented by larger, lighter colored symbols, consistent with acquired resistance mediated by *ABCB1* overexpression as a consequence of the selective pressure applied by *in vivo* exposure of repeated doses of taxol chemotherapy. Interestingly, OCMs located in the top right sector (e.g., 130, 161-1, and 202-3) were represented by small, darker colored symbols (Figure 6), likely reflecting tumors with a high degree of intrinsic taxol resistance. By contrast, OCMs in the lower left sector (e.g., 361a, 341-1, and 376a) likely represent intrinsically sensitive cancers.

With the possible exception of 86 and 202-3, there was little evidence of MDR1i-mediated desensitization, consistent with the therapeutic paradigm that inhibiting efflux pumps should only induce chemotherapy sensitization. A few other exceptions warrant discussion: OCMs 64-1, 165, 293-4, and 320 sit in the top right quadrant and are thus relatively resistant to taxol. They lie near the linear regression line, i.e., they are not re-sensitized by the MDR1i. Despite this, they overexpress *ABCB1* and are derived from patients with high taxol exposure (Figure 6). *ABCB1* upregulation may have played a role in taxol resistance at some point in the tumor’s evolutionary trajectory, but then additional drug resistance mechanisms became more dominant, such that inhibiting MDR1 in OCM is not sufficient to re-sensitize. OCM.74-3 is also interesting in this context; like OCMs 74-1 and 74-5, it is derived from a patient with high taxol exposure and is an *ABCB1* super-expresser (Figure 6). Yet, it is relatively taxol sensitive (Figure S2c). One possibility is that while *ABCB1* upregulation is an important mediator of taxol resistance in this tumor, in the subclone represented by this particular OCM, this mechanism has been subverted by another adaptation that offers a proliferative advantage. Presumably, further exposure to taxol would suppress this subclone and allow those represented by OCMs 74-1 and 74-5 to expand. 341-3 also stands out; it has high *ABCB1* expression despite low taxol exposure and is not sensitized by MDR1i. Whether the OCM expresses functional MDR1 remains to be determined. Nevertheless, this integrated analysis identifies a subset of at least eight OCMs that reflect acquired taxol resistance mediated by *ABCB1* overexpression in response to accumulated taxol chemotherapy.

### OCMs re-sensitized to taxol via the MDR1i combination are sensitive to cabazitaxel monotherapy

Our observations are consistent with prior studies and confirm that drug efflux mechanisms are a major contributor of taxol resistance in a subset of HGSOCs.^23^^,^^24^ While efflux inhibitors have been explored as chemotherapy sensitizers, enthusiasm for this approach has waned.^99^ Therefore, an alternative approach to tackle MDR1i-mediated resistance is deployment of agents that are poor substrates of *ABC*-family member transporters. Cabazitaxel is a semisynthetic taxane with poor affinity for MDR1 compared with taxol and is approved for use in the treatment of patients with hormone-refractory metastatic prostate cancer.^100^^,^^101^ However, its clinical use for ovarian cancer has not been extensively explored.^102^

**Figure 7:**
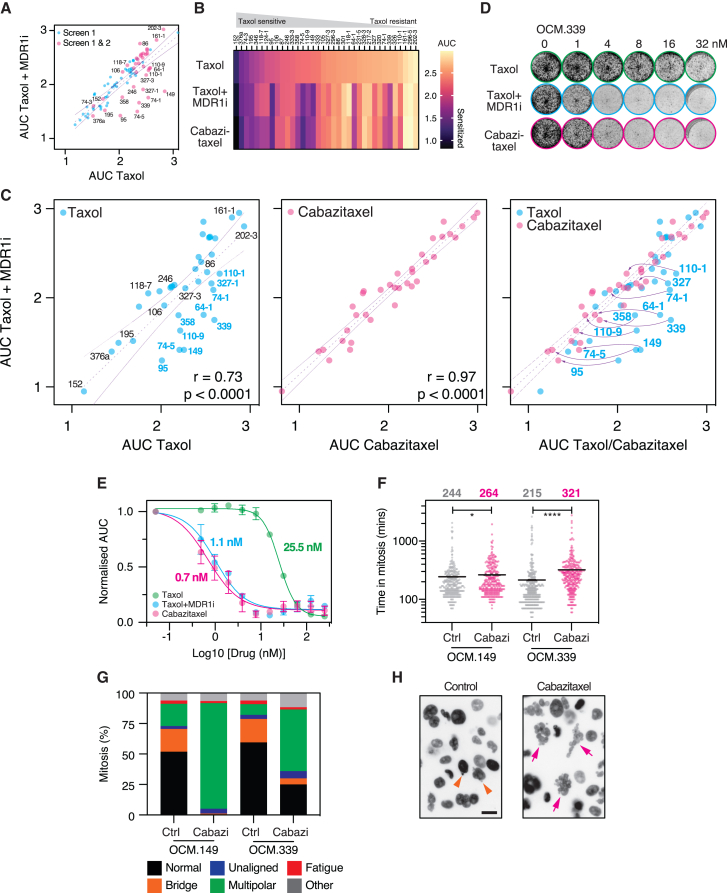
OCMs re-sensitized to taxol via MDR1i are sensitive to cabazitaxel monotherapy (A) *xy* graph plotting AUC values for taxol against taxol + MDR1i, as shown in Figure 2C, highlighting 36 OCMs screened with cabazitaxel. (B) Heatmap showing AUC values of OCMs rescreened with taxol, taxol + MDR1i, or cabazitaxel, rank ordered by Taxol response. Data are average from three biological replicates. (C) *xy* graphs plotting AUC values for taxol + MDR1i against either Taxol (left) or cabazitaxel (middle). Superimposing these two graphs (right) highlights ten OCMs (blue) sensitive to cabazitaxel monotherapy. Lines show linear regression with 95% confidence intervals and the values represent Spearman r correlations; *p* < 0.0001 (left and center). (D) CFA of OCM.339 after exposure to taxol ± MDR1i or cabazitaxel. (E) Dose-response curves for OCM.149 exposed to taxol ± MDR1i or cabazitaxel with proliferation measured by time-lapse imaging of cells expressing GFP-H2B, imaging every 4 h for at least 144 h. Graphs show the mean and SEM from at least three biological replicates. Values show GI_50_. (F) Time-lapse analysis of GFP-H2B-expressing OCMs 149 and 339, untreated (Ctrl) or exposed to 4 nM cabazitaxel (Cabazi). Time in mitosis represents nuclear envelope breakdown (NEBD) to anaphase onset, with the mean indicated. At least 250 cells analyzed per condition. Mann-Whitney test ∗*p* < 0.05, ∗∗∗∗*p* < 0.0001. (G) Bar graph quantifying mitotic abnormalities. (H) Images of GFP-H2B-expressing OCM.149 after 72 h, showing micronuclei (arrow heads) in control cells and highly abnormal nuclei (arrows) in cabazitaxel-treated cells. Bar 20 μm. Data in (F)–(H) derived from one biological replicate. See Figure S10.

To explore the potential of cabazitaxel, we re-screened 36 OCMs, focusing on those sensitized by the MDR1i, a selection of taxol-resistant OCMs, and four relatively sensitive ones (Figure 7A). As aforementioned, each OCM was subjected to a mini-titration of taxol alone, taxol plus MDR1i, and cabazitaxel alone. Importantly, there were strong correlations between the two screens (Figure S10A). Interestingly, an AUC heatmap indicated that if an OCM was re-sensitized to taxol by the MDR1i, it was also sensitive to cabazitaxel (Figure 7B). To test this more rigorously, we plotted the AUC for taxol/MDR1i against AUC for taxol or cabazitaxel alone (Figure 7C). As before, there was a strong correlation between taxol/MDR1i and taxol, but with the known MDR1i responders falling below the linear regression. Strikingly, the correlation between taxol/MDR1i and cabazitaxel was even stronger, indicating that each OCM was equally sensitive to the two treatments. Superimposing the two graphs showed that the increased correlation was largely driven by 10 OCMs moving to the left on the *x* axis. This included seven of the eight OCMs previously identified as clear MDR1i responders, including 149, 339, and 74-1, and three that were previously borderline responders, 110-1/9 and 64-1 (Figures S1 and S10B). MDR1i non-responders were not sensitive to cabazitaxel, indicating that cabazitaxel only reverts acquired taxol resistance mediated by drug efflux mechanisms. Interestingly, OCM.246 was not sensitized by the MDR1i in this second screen. One possibility is that during continued *ex vivo* cell culture, *ABCB1* overexpression has been lost. Nevertheless, a subset of OCMs that can be re-sensitized to taxol via combination with the MDR1 inhibitor are sensitive to cabazitaxel monotherapy.

Interrogation of CFA images and additional 10-point dose response confirmed that OCMs sensitive to taxol/MDR1i were also sensitive to cabazitaxel, while OCMs not re-sensitized by MDR1i were not sensitive to cabazitaxel (Figures 7D, S10C, and S10D). For OCM.149, the MDR1i combination and cabazitaxel had very similar effects, reducing the GI_50_ values from over 25 nM to approximately 1 nM (Figure 7E). To confirm that cabazitaxel sensitivity was via expected mitotic mechanisms, we analyzed OCMs 149 and 339 by time-lapse microscopy. In both cases, cabazitaxel delayed mitotic progression (Figure 7F), albeit modestly in the case of 149, and induced a marked increase in multipolar divisions (Figure 7G). Inspection of image sequences revealed micronuclei in untreated OCM.149, consistent with persistent CIN, while exposure to cabazitaxel showed a marked increase in nuclei with a *bunch of grapes* morphology (Figure 7H), indicating multi-nucleation following highly catastrophic mitoses. Thus, cabazitaxel toxicity in taxol-resistant HGSOC involves a mitotic delay followed by catastrophic multipolar divisions.

## Discussion

To address clinical challenges with taxol resistance and toxicity, significant human endeavor has been directed at understanding taxol’s mode of action and developing targeted drugs against key mitotic regulators or apoptotic pathways activated by abnormal mitosis. While numerous drugs and combination strategies have been explored in various model systems, a limitation has been the availability of a platform to systematically compare new strategies. To address this, we analyzed 83 patient-derived *ex vivo* cell culture models that capture the extensive intra- and inter-patient heterogeneity typical of HGSOC.^37^^,^^89^ We evaluated—in parallel—four approaches to modulate taxol sensitivity.

The HSETi combination did not have a major impact on the taxol sensitivity landscape. This is surprising because centrosome amplification has been described in HGSOC,^38^^,^^39^^,^^40^ OCMs often undergo multipolar mitosis,^37^ and we observed centrosome de-clustering with the HSETi. However, higher AZ82 concentrations did enhance OCM.149 taxol sensitivity, suggesting that inhibition of HSET may be effective in a small subset of HGSOC. Exploring this further will benefit from a more potent range of HSET inhibitors. Indeed, HSET continues to emerge in genetic screens for tumor cell vulnerabilities,^103^^,^^104^ supporting the original hypothesis that suppressing spindle pole clustering in centrosome-amplified cancer cells suppresses proliferative potential.

In contrast, inhibiting Mps1 did influence the taxol sensitivity landscape, sensitizing and desensitizing a number of OCMs. Interestingly, sensitization was accompanied by an increase in multipolar mitoses. Moreover, when OCMs overexpressing *ABCB1* were re-sensitized to low-nanomolar concentrations of taxol, the frequency of multipolar mitoses increased. These observations indicate that, as in breast cancer,^21^^,^^22^ multipolar mitoses are a major driver of taxol toxicity in ovarian cancer. This compounds our surprise regarding the lack of an HSETi effect. Nevertheless, because multipolar mitoses correlate with taxol sensitization induced by both the Mps1i and MDR1i, our observations support taxol’s toxicity occurring via a mitotic phenomenon, rather than disrupting microtubule functions in interphase. Moreover, while the tumor microenvironment may play a role in eliminating tumor cells following an abnormal mitosis,^105^ the fact that *ABCB1* overexpression correlates with acquired taxol resistance indicates that taxol’s primary target is the tumor cell, not the microenvironment, at least in this disease. While a mechanism to explain the Mps1i sensitization effect may be emerging, translating this into a clinical strategy will be challenging, particularly as Mps1i desensitized some OCMs to taxol, as seen previously,^22^^,^^63^^,^^106^^,^^107^^,^^108^ and there is no predictive biomarker. We note that several OCMs sensitized by Mps1i were also MDR1i responders. The Mps1i used here is not an MDR1 substrate because it blocked kinetochore recruitment of O-Mad2 in OCMs 74-1 and 339, which overexpress *ABCB1* and are MDRi responders. Rather, inhibiting the SAC probably lowers the effective intra-cellular taxol threshold. Targeting the SAC might therefore be a promising approach where acquired resistance is driven by drug efflux. However, whether this would offer any advantages over targeting drug efflux directly remains to be seen.

The screen indicated that inhibiting Bcl-xL had a wide-ranging effect on the taxol sensitivity landscape, with the vast majority being sensitized. However, closer inspection indicated that this was driven by Bcl-xLi monotherapy, rather than the combination, suggesting that OCMs are already under sufficient stress that additional chemotherapy-induced stress is not required to induce apoptosis. The drivers of this pre-existing stress are unclear but could be multiple factors. Oncogenic signaling is a known driver of apoptosis that is buffered during tumorigenesis by suppressing apoptosis.^109^ Correlating Bcl-xLi sensitivity with oncogenic signaling is an important next step, which may be particularly important when comparing epithelial ovarian cancer subtypes. While the screen was dominated by HGSOC-derived OCMs, the seven OCMs derived from four patients with LGSOC were also sensitive to the Bcl-xLi. Hallmarks of HGSOC are amplification of *MYC* or *CCNE1*,^110^ while LGSOC are characterized by mutations in the RAS-MAPK-MEK pathway.^111^^,^^112^ Indeed, mutant RAS signaling may upregulate Bcl-xL via STAT3 to suppress apoptosis, leading to Bcl-xLi sensitivity.^113^ Since MYC can also modulate apoptotic responses,^63^^,^^114^^,^^115^ comparing Bcl-xLi sensitivity in MYC-driven and non-MYC-driven ovarian cancers will be important.

An alternative source of endogenous stress could be the prolonged mitoses observed in OCMs, a hypothesis that stemmed from our previous studies.^63^^,^^70^^,^^75^ However, we did not observe an obvious correlation between mitotic duration and Bcl-xLi-sensitivity, in line with a recent finding that apoptotic priming induced by centrosome amplification was not related to mitotic duration.^40^ However, our hypothesis was based on analyzing near-diploid, karyotypically stable colon cancer cell lines where the overlapping functions of Mcl-1 and Bcl-xL contribute to pro-survival activity.^63^^,^^70^ That many OCMs are sensitive to Bcl-xLi monotherapy suggests that Mcl-1 is not a ubiquitous pro-survival factor in this context. Thus, rather than protracted mitoses, the high levels of CIN displayed by the OCMs may drive other forms of stress leading to apoptotic priming, such as centrosome amplification,^40^ or proteotoxic and autophagic stress.^116^

While the exact mechanisms responsible for the Bcl-xLi monotherapy effect remain to be determined, our observations highlight the potential of Bcl-xL as a target in both HGSOC and LGSOC. This is particularly important for LGSOC where standard chemotherapy is not effective.^90^^,^^91^ However, translating Bcl-xL inhibitors into the clinic is challenging: the BH3 mimetics analyzed thus far induce thrombocytopenia due to Bcl-xL’s role in maintaining platelet lifespan.^117^ To address this, strategies to specifically target Bcl-xL in tumor cells are being explored, including proteolysis targeting chimeras (PROTACs) and antibody-drug conjugates (ADCs).^118^ An important next step therefore is to screen a diverse panel of OCMs, comparing a BH3 mimetic with PROTAC- and/or ADC-based therapies.

Drug efflux mechanisms have emerged as a major contributor to the acquired chemotherapy resistance of HGSOC, driven in large part by chromosomal translocations leading to rearrangements of *ABCB1.*^23^^,^^24^ While cisplatin and carboplatin are poor MDR1 substrates, taxol, docetaxel, topotecan, and doxorubicin, all of which are used to treat ovarian cancer, are readily exported, leading to the concept of multi-drug resistance. Whether taxol exposure of any given patient was the selective pressure leading to the emergence of *ABCB1* overexpression is unclear. For example, patient 74 was exposed to multiple doses of pegylated liposomal doxorubicin (PLD; Caelyx) in addition to taxol prior to biopsy. By contrast, patient 327 was exposed to multiple cycles of taxol but only one cycle of PLD prior to sampling. Patient 110 was only exposed to carboplatin and taxol, suggesting that taxol provided the relevant *in vivo* selective pressure leading to MDR1-dependent taxol resistance in OCM.110-9. However, OCM.110-1 generated from a biopsy collected prior to chemotherapy also overexpresses *ABCB1* and is sensitized to taxol by MDR1i. This suggests that *in vivo* selective pressures other than chemotherapy can upregulate efflux mechanisms causing intrinsic resistance.

Regardless of the selective pressures that result in the expansion of subclones overexpressing *ABCB1*, our observations confirm prior studies showing that inhibition of MDR1 restores taxol sensitivity.^24^^,^^67^ In light of the limited treatment options for platinum-resistant HGSOC, and the widespread use of taxol in this disease, exploiting this mechanism presents a therapeutic strategy that warrants further exploration. However, because early clinical studies evaluating drug efflux inhibitors were unsuccessful, enthusiasm for revisiting MDR1 inhibitors is limited, despite the well-articulated limitations of these early trials.^88^ An alternative approach is to deploy a taxane that is not an MDR1 substrate. Here, we show an almost perfect correlation between sensitivity to the taxol-MDR1i combination and cabazitaxel. Because cabazitaxel is well studied in the context of prostate cancer,^100^ our observations make a compelling case to explore this drug in taxol-resistant HGSOC. An important next step therefore is to develop a biomarker to determine which patients to treat with cabazitaxel and when. Although the interrogation of OCMs, which are highly purified tumor fractions, can detect both *ABCB1* fusions and MDR1 overexpression (study by Williams et al.^119^ and this study), the OCM pipeline is not yet suitable for functional interrogation in real time to support clinical decision-making. Therefore, it will be important to adapt next-generation sequencing platforms to screen crude biopsies. Also, it will be important to screen for *ABCB1*-mediated resistance when acquired taxol resistance emerges, i.e., interrogating primary surgical samples is unlikely to be informative in the majority of cases. However, the OCM pipeline does show that the ability to collect ascites upon disease recurrence provides an opportunity to identify cancers with acquired taxol resistance that are cabazitaxel sensitive.

### Limitations of the study

Throughout, drug sensitivity was assayed in 2D cultures of purified tumor cells. Therefore, we have not accounted for how a 3D microenvironment and/or the presence of stromal cells may influence outcome. OCMs can be cultured in 3D and as co-cultures,^37^ so future experiments will be able to address this. Another important area for future focus will be the development of predictive biomarkers. While *ABCB1* overexpression correlates with cabazitaxel sensitivity, we have not yet validated its predictive potential. Thus, exploring *ABCB1* as a predictor of cabazitaxel sensitivity, following acquired taxol resistance in a separate cohort of OCMs, and developing a clinically deployable biomarker will be important next steps.

## Resource availability

### Lead contact

Further information and reagent requests may be directed to the lead contact, Stephen S. Taylor (stephen.taylor@manchester.ac.uk).

### Materials availability

All unique/stable reagents generated in this study will be made available on request, but we may require a payment and/or a completed materials transfer agreement, in particular if there is potential for commercial application.

### Data and code availability

• RNA-seq data from 45 previously published OCMs^37^^,^^120^^,^^121^^,^^122^ and 33 OCMs new to this study are available from EMBL-EBI (EMBL-EBI: E-MTAB-7223, E-MTAB-10801, E-MTAB-11000, E-MTAB-14568) and are publicly available as of the date of publication. *ABCB1* expression levels are in Table S2. Original images and microscopy data reported in this paper, and any additional information required to reanalyze the data, will be shared by the lead contact upon request.
• The paper contains no original code.
• Any additional information required to reanalyze the data reported in this paper is available from the lead contact upon request.

## Acknowledgments

This work was supported by 10.13039/501100000289Cancer Research UK (C1422/A31334, C147/A25254, and C19941/A28707; RCCPOB-May23/100005), the 10.13039/501100000272National Institute for Health and Care Research (CL-2022-06-002; NIHR203308), the 10.13039/501100000265Medical Research Council (MR/X008088/1), and the 2022–25 UKRI MRC Impact Accelerator Account (University of Manchester, MR/X502868/1) awarded by the Translation Manchester Confidence for Translation scheme. The views expressed are those of the author(s) and not necessarily those of the NIHR or the Department of Health and Social Care.

We thank the patients for their commitment to research, the MCRC Biobank for the sample collection, the Genomic Technologies Core Facility at The University of Manchester for performing the RNA sequencing, and members of the Taylor lab for advice and comments on the manuscript.

## Author contributions

Methodology, A.T., L.N., R.D.M., B.M.B., I.-H.L., S.L., and J.A.; investigation, A.T., L.N., R.D.M., B.M.B., and S.L.; data curation, A.T., R.D.M., B.M.B., I.-H.L., and J.C.M.; validation, A.T.; formal analysis, A.T., R.D.M., B.M.B., and S.S.T.; conceptualization, S.S.T.; funding acquisition, R.D.M., B.M.B., J.C.M., and S.S.T.; supervision, S.S.T.; project administration, J.C.M., J.A., and S.L.; visualization, A.T. and S.S.T.; writing – original draft, A.T., B.M.B., I.-H.L., J.L.T., J.C.M., and S.S.T.; writing (review and editing), all authors. All authors read and approved the final manuscript.

## Declaration of interests

The authors declare no competing interests.

## STAR★Methods

### Key resources table

**Table:** 

REAGENT or RESOURCE	SOURCE	IDENTIFIER
**Antibodies**		

Sheep polyclonal anti-Aurora A	Girdler et al. 2006^123^	N/A
Sheep polyclonal anti-Mad2	Johnson et al. 2004^124^	N/A
Rabbit anti-Phospho-Histone H3 Ser10	Merck Millipore	Cat#06–570; RRID: AB_310177
Rabbit anti-Pericentrin	AbCam	Cat#ab4448; RRID: AB_304461
Rabbit anti-MDR1	Proteintech	Cat#22336-1-AP; RRID: AB_2833023
Human anti-ACA	Earnshaw et al. 1986^125^	N/A
Mouse anti-p53 (DO-1)	Santa Cruz Biotechnology	Cat#sc-126; RRID: AB_628082
Donkey anti-Sheep Cy3	Jackson ImmunoResearch Laboratories Inc	Cat#713-165-147; RRID: AB_2315778
Donkey anti-Sheep Cy2	Jackson ImmunoResearch Laboratories Inc	Cat#713-225-147; RRID:AB_2340735
Donkey anti-Rabbit Cy3	Jackson ImmunoResearch Laboratories Inc	Cat#711-165-152; RRID: AB_2307443
Donkey anti-Human Cy2	Jackson ImmunoResearch Laboratories Inc	Cat#709-225-149; RRID: AB_2340541
Donkey anti-Mouse Cy3	Jackson ImmunoResearch Laboratories Inc	Cat#715-165-150: RRID: AB_2340813
Mouse monoclonal anti-Myc-tag	Merck Millipore	Cat#05–724; RRID: AB_309938
Sheep polyclonal anti-Bub3	A.J. Holland and S.S. Taylor, unpublished	N/A
Rabbit anti-MDR1	Proteintech	Cat#22336-1-AP; RRID: AB_2833023
Rabbit anti-MDR1(D3H1Q)	Cell Signaling Technology	Cat#12683; RRID: AB_2715689
Rabbit anti-Sheep IgG (HL) HRP	Invitrogen	Cat#G21040; RRID: AB_2536527
Goat anti-Mouse IgG (HL) HRP	Invitrogen	Cat#G21234 RRID: AB_2536530
Goat anti-Rabbit IgG (HL) HRP	Merck Millipore	Cat#ABC240; RRID: AB_2722647

**Bacterial and virus strains**		

XL1-Blue competent cells	Agilent Technologies	Cat#200249

**Biological samples**		

Patient Samples	MCRC Biobank Manchester	N/A

**Chemicals, peptides, and recombinant proteins**		

Nutlin-3	Sigma-Aldrich	Cat#N6287
Nocodazole	Sigma-Aldrich	Cat#M1404
AZ3146 (Mps1i)	Selleckchem	Cat#S2731
Hoechst 33358	Sigma-Aldrich	Cat#B1155
Taxol	Sigma-Aldrich	Cat#T7402
Carboplatin	Selleckchem	Cat#S1215
Elacridar (MDR1i)	Selleckchem	Cat#S7772
A-1155463 (Bcl-xLi)	Selleckchem	Cat#E2926
AZ82 (HSETi)	Sigma-Aldrich	Cat#533916
Cabazitaxel	Selleckchem	Cat#S3022
Puromycin	Sigma-Aldrich	Cat#P7255
Hygromycin	Roche	Cat#10843555001
Blasticidin S Hydrochloride solution	Melford	Cat#B12150–0.1
Tetracycline hydrochloride	Sigma-Aldrich	Cat#T7660
Dulbecco’s Modified Eagle Medium (DMEM)	Life Technologies	Cat#41966052
RPMI 1640 Medium	Life Technologies	Cat#21875034
OCMI (also available from USBiological #506390)	Ince et al. 2015^126^	N/A

**Critical commercial assays**		

ProFection® Mammalian Transfection System	Promega	Cat#E1200
cobas® DNA Sample Preparation Kit	Roche	Cat#05985536190
TruSeq® DNA PCR-Free Kit	Illumina Inc	Cat#20015962
Stranded mRNA Prep Ligation kit	Illumina Inc	Cat# 20040532
RNeasy Plus Mini Kit	Qiagen	Cat#74134
QIAprep Spin Miniprep Kit	Qiagen	Cat#27104
SuperscriptTM III One-Step RT-PCR Platinum Taq HiFi	Thermo Fisher Scientific	Cat#12574035
Tumor Dissociation kit	Miltenyi Biotec	Cat#130095929

**Deposited data**		

RNA sequencing of 78 ovarian cancer models (45 published previously)	Nelson et al. 2020^37^; Barnes et al. 2021^120^; Coulson-Gilmer et al. 2021,^121^ Littler et al. 2025.^122^ EBML-EBI, European Nucleotide Archive	EMBL-EBI: E-MTAB-7223;EMBL-EBI: E-MTAB-10801;EMBL-EBI: E-MTAB-11000;EMBL-EBI: E-MTAB-14568

**Experimental models: Cell lines**		

AAV293T	Agilent Technologies	Cat#240073
BT549	ATCC	Cat#HTB-122; RRID: CVCL_1092
RKO Flp-In™T-Rex™	Topham et al. 2015^63^	N/A
RKO/FRT/TO/Myc-BCL-xL	Topham et al. 2015^63^	N/A
RKO/FRT/TO/Myc-MDR1	This study	N/A

**Oligonucleotides**		

Primer: XhoI *TP53* 5′- CACCTCGAGGAGGAGCCGCAGTC AGATCCTA	Invitrogen	N/A
Primer: NotI *TP53* 3′- CACGCGGCCGCTCACAGTCTGAG TCAGGCCCTTCTGTC	Invitrogen	N/A
*TP53* sequencing primers: 5-CACCAGCAGCTCCTACACCG-3′ 5′-ATGAGCGCTGCTCAGATAGCG-3′	Invitrogen	N/A
*TP53* sequencing primers: 5-CGGCTCATAGGGCACCACC-3′ 5-TCTTCTTTGGCTGGGGAGAGG-3′	Invitrogen	N/A
Primer: XhoI Mdr1 5′-CACCTCGAGGATCTTGAAGGGGAC CGCAATG	Invitrogen	N/A
Primer: NotI Mdr1 3′-CACGCGGCCGCTCACTGGCGCTT TGTTCCAG	Invitrogen	N/A

**Recombinant DNA**		

pHaMDRwt	Pastan et al. 1988^127^	RRID: Addgene_10957
psPAX2	Didier Trono unpublished	RRID: Addgene_12260
pMD2.G	Didier Trono unpublished	RRID: Addgene_12259
pOG44	ThermoFisher Scientific	Cat#V600520
pLVX-myc-EmGFP-H2B	Pillay et al. 2019^128^	N/A
pcDNA5/FRT/TO/Myc-epitope tag	Girdler et al. 2006^123^	N/A
pBluescript SK-vector	Agilent Technologies	Cat#212206

**Software and algorithms**		

MetaMorph® Microscopy Automation & Image Analysis Software	MDS Analytical Technologies	RRID:SCR_002368
Adobe Photoshop® CC 2024	Adobe Systems Inc	RRID:SCR_014199
Prism10	GraphPad	RRID:SCR_002798
IncuCyte S3 Live Cell Analysis System	Sartorius	RRID:SCR_023147
Seqman Pro (DNASTAR)	Lasergene Core Suite	RRID:SCR_000291
bcl2fastq	Illumina Inc	RRID:SCR_015058;https://support.illumina.com/sequencing/sequencing_software/bcl2fastq-conversion-software.html
FastQC	Babraham Bioinformatics	RRID:SCR_014583; https://www.bioinformatics.babraham.ac.uk/projects/fastqc/
FastQ Screen	Babraham Bioinformatics	RRID:SCR_000141; https://www.bioinformatics.babraham.ac.uk/projects/fastq_screen/
Bestus Bioinformaticus Duk (BBDuk)	Bestus Bioinformaticus	RRID:SCR_016969; https://sourceforge.net/projects/bbmap/
STAR	Dobin et al. 2013^129^	RRID:SCR_004463
SynergyFinder+ web application	Zheng et al. 2022^130^	https://synergyfinder.org/
R package DESeq2	Bioconductor	RRID:SCR_015687; https://bioconductor.org/packages/release/bioc/html/DESeq2.html

**Other**		

Q5® High-Fidelity DNA Polymerase	New England Biolabs	Cat#M0491S
Lipofectamine	Thermo Fisher Scientific	Cat#18324012
PLUS™ Reagent	Thermo Fisher Scientific	Cat#11514015
IncuCyte® Caspase-3/7 Dye for Apoptosis Reagent	Sartorius	Cat#4440

### Experimental model and study participant details

#### Patient sample collection

Research samples were obtained with informed patient consent from the Manchester Cancer Research Center (MCRC) Biobank (Human Tissue Authority license: 30004), which is ethically approved as a research tissue bank by the South Manchester Research Ethics Committee (ref. 22/NW/0237). The role of the MCRC Biobank is to distribute samples; it does not endorse studies performed or the interpretation of results. For more information, see https://www.mcrc.manchester.ac.uk/research/mcrc-biobank. All patients were women diagnosed with epithelial ovarian cancer as described in Table S1, with relevant treatments indicated in Figures S1, S9 and S10.

#### *Ex vivo* ovarian cancer models

Eighty-three OCMs from 68 patients (age: 25–84 years) were generated from ascitic fluid or solid tumor samples, 45 of which are published previously (Table S1).^37^^,^^120^^,^^121^^,^^122^^,^^128^^,^^131^^,^^132^ To establish OCMs first described here,^37^ ascites were centrifuged, red blood cells removed and remaining cells plated into Primaria or Cell+ flasks containing OCMI.^126^ Solid tumor samples were processed using a tumor dissociation kit (Miltenyi Biotec) and cells plated into collagen-coated flasks containing OCMI. Cultures were incubated at 37°C for 2–4 days in a humidified 5% CO_2_ and 5% O_2_ atmosphere, then media replaced every 3–4 days. Once attached, selective trypsinisation was used to separate stromal and tumor cells. Established OCMs were cultured in OCMI.^37^^,^^89^^,^^126^ In 55 cases, each patient is represented by a single OCM. However, 13 patients are represented by 28 OCMs due to longitudinal sampling or spatially resolved biopsies (Table S1 and Figure S1), creating 13 subsets. These subsets include: 10 longitudinal pairs (e.g., 66-1 and 5); one pair generated from spatially resolved solid samples collected at the same timepoint (361a and 361b); and triplet sets from two patients, 64 and 74 (Figure S1). The set from patient 64 included a longitudinal pair, OCMs 64-1 and 64-3, with the latter harboring two distinct subclones with differential EpCAM status, 64-3-Ep+ and 64-3-Ep-.^37^^,^^89^ These two subclones harbor the same *TP53* mutation (Table S1), but differ in terms of nuclear atypia, karyotypes and several tumor markers.^37^^,^^89^

#### Cell lines

Flp-In T-Rex RKO cells,^63^ RKO/FRT/TO/Myc-Bcl-xL,^63^ RKO/FRT/TO/Myc-MDR1 (this study) and AAV293T (Agilent Technologies) cells were cultured in Dulbecco’s Modified Eagle Medium (DMEM); BT549 cells (ATCC; RRID: CVCL_1092) were cultured in RPMI-1640 media; both supplemented with 10% fetal bovine serum (FBS), 100 U/ml penicillin, 100 μg/mL streptomycin and 2 mM glutamine and maintained at 37°C in a humidified 5% CO_2_ atmosphere. Cells were periodically authenticated (Promega Powerplex 21 System) and tested for the presence of mycoplasma by the Molecular Biology Core Facility at the CRUK Manchester Institute.

### Method details

#### DNA sequencing

##### *TP53* genotyping of primary tumors

Archival FFPE tumor blocks were retrieved by the MCRC Biobank for genotyping by Manchester Center for Genomic Medicine, St Mary’s Hospital, Manchester (Table S1). As described previously,^37^ FFPE blocks were assessed for total cellularity and neoplastic cell content (percentage of all nucleated cells on a Haematoxylin and Eosin-stained slide). A neoplastic cell count of ≥10% was required. Tumor from 5 × 5 μM unstained pathology slides was available for DNA extraction using the cobas DNA Sample Preparation Kit (Roche), before DNA quantification using a Qubit 2.0 Fluorometer (ThermoScientific). Targeted enrichment was performed using the GeneRead Clinically Relevant Tumor Targeted Panel V2 (Qiagen). Library preparation was performed using the TruSeq DNA PCR-Free Kit (Illumina). Next generation sequencing was performed on an Illumina MiSeq platform using 2 x 150 paired-end sequencing chemistry. For somatic variants the target read depth across all coding regions (exon 2 to 9) was 350× minimum. Mutations were named according to Human Genome Variation Society guidelines (http://www.hgvs.org/) using reference sequence NM_000546.5. All variant calls were independently reviewed using the BAM files and a genome browser (Integrated Genomic Viewer). At a variant allele frequency ≥4% the call sensitivity was >90% and specificity >95% after manual review.

##### OCM *TP53* genotyping

RNA was extracted using RNeasy Plus Mini kit (Qiagen) and *TP53* complementary DNA generated using Superscript III One-Step RT-PCR Platinum Taq HiFi (Thermofisher) and primers 5′-CACCTCGAGGAGGAGCCGCAGTCAGATCCTA; 3′-CACGCGGCCGCTCACAGTCTGAGTCAGGCCCTTCTGTC. PCR products were cloned into a pBluescript SK-vector, transformed into XL1-Blue competent cells, plasmid DNA extracted using QIAprep Spin Miniprep Kit (Qiagen) and sequenced using primers: 5′-CACCAGCAGCTCCTACACCG-3′, 5′-ATGAGCGCTGCTCAGATAGCG-3', 5′-CGGCTCATAGGGCACCACC-3′, 5′-TCTTCTTTGGCTGGGGAGAGG-3′. Sequences were aligned using Seqman Pro (DNASTAR). For OCMs first described here the pBluescript-p53 vectors were subjected to whole-plasmid sequencing by nanopore (Plasmidsaurus).

#### Drug concentrations

The drug concentrations in this study were selected based on several factors including consideration of the literature, previous results from our laboratory, pilot studies empirically testing a range of concentrations, and interrogation of pharmacodynamic biomarkers. For the HSET inhibitor (AZ82; Sigma-Aldrich) we selected an initial concentration of 2 μM based on the analysis by Wu et al., 2013.^92^ Consistent with these observations, 2 μM de-clustered extra nummary spindle poles in BT549 cells (Figure S4A). While we used 2 μM in the screen, we also tested a subset of OCMs at higher concentrations, up to 16 μM (Figure S4D). The Mps1 inhibitor (AZ3146; Selleckchem) was used at 2 μM based on Hewitt et al. 2010.^93^ Immunofluorescence analysis of OCM.74-1 showed that this concentration suppressed kinetochore recruitment O-Mad2 (Figure 3A), consistent with on-target activity. Furthermore, this concentration accelerated progression through mitosis, consistent with SAC attenuation (Figure 3E). The Bcl-xL inhibitor (A-1155463; Selleckchem) was used in the screen at 100 nM based on empirical evidence. In brief, RKO cells harboring a tet-inducible Bcl-xL transgene^63^ were exposed to taxol to induce apoptosis. This was then reverted by tet-induction of Bcl-xL. The Bcl-xLi was then titrated from 1, 10, 25, 50, 100 and 500 nM to determine which concentrations suppressed the survival-promoting effect of the transgene. 100 nM restored cell death but did not overshoot the apoptosis-inducing effect of the taxol (Figure 4A). That 100 nM of A-1155463 is a discerning concentration, not a blanket toxic dose, is consistent with prior observations, e.g., Iavarone et al., 2019.^82^ The MDR1 inhibitor Elacridar (Selleckchem) was initially used at 250 nM based on Christie et al., 2019,^24^ and empirical evaluation of an RKO cell line expressing a tet-inducible MDR1 transgene (Figure 5A and S8A–C). In addition, we empirically tested the MDR1i at 1, 10, 25, 50, 100, 250 (Figure S7B) and 2, 4, 8, 15, 31, 62, 125 and 250 nM (Figure S8E).

#### Drug-sensitivity screens

All drugs were dissolved in DMSO except carboplatin, which was dissolved in PBS. All agents were aliquoted and stored at −80°C. Combination colony formation assays (CFA) were performed in parallel with taxol and carboplatin mini-titrations. Each CFA included two technical replicates for taxol and taxol plus elacridar, and each assay was performed in triplicate, yielding: six mini-titrations for taxol and taxol plus elacridar; and three for either carboplatin, taxol plus Mps1, taxol plus HSET or taxol plus Bcl-xL inhibitor.

Cells were seeded at 1–3 x 10^4^ cells/well into 24-well Primaria plates. The following day a titration of taxol (0, 1, 4, 8, 16, 32 nM; Sigma-Aldrich) with or without the addition of either 250 nM elacridar, 2 μM Mps1 inhibitor, 100 nM Bcl-xL inhibitor or 2 μM HSET inhibitor, or a titration of carboplatin (0, 1, 10, 25, 50, 100 μM; Selleckchem) alone was added to the cells. 36 OCMs were included in a second screen with mini-titrations of taxol (0, 1, 4, 8, 16, 32 nM; Sigma-Aldrich), with or without 250 nM elacridar, and cabazitaxel (0, 1, 4, 8, 16, 32 nM; Selleckchem) in triplicate. Cells were incubated with the agents for 6 days, then fixed in 1% formaldehyde, before being stained with a 0.05% v/v crystal violet (Sigma Aldrich) solution. Additional titrations of HSETi, Bcl-xLi and MDRi for synergy analyses followed the same protocol. For the 10-point dose-response assays of taxol (0, 0.5, 1, 2, 4, 8, 15, 31, 62, 125, 250 nM; Sigma-Aldrich) with or without 250 nM elacridar (Figure 5C), cells were re-exposed, and the plates fixed and stained after 14 days for OCM.246 and after 17 days for OCM.149. For CFA using RKO/FRT/TO/Myc-Mdr1 (Figures 5A and S8), cells were plated in the presence or absence of 1 μg/mL tetracycline. The following day a titration of taxol (0, 3.12, 6.25, 12.5, 25, 50 nM) with or without 250 nM elacridar, with or without 1 μg/mL tetracycline, was added. After 72 h the plates were fixed and stained as described above.

Stained plates were imaged on a ChemiDoc Touch Imaging System (BioRad) before the crystal violet was extracted with 10% acetic acid. The absorbance at 570 nm was read on a VarioSkanLUX multimode microplate plate reader (Thermo Scientific). The mean absorbance from six 10% acetic acid-only blank wells was subtracted from each value, before normalisation to the untreated control well. Averages from at least three biological replicates were used to generate GI _50_ curves and Area Under the Curve (AUC) values using Prism 10 (GraphPad) software. Note that the term GI_50_ refers to the concentration that yields 50% of maximal inhibition of growth. Synergy analysis was performed via the SynergyFinder+ web application (www.synergyfinder.org, 07.09.2024-R-3.10.3).^130^^,^^133^

#### Generation of RKO Flp-In T-Rex MDR1 cells

The *ABCB1* open reading frame was PCR-amplified from plasmid pHaMDRwt (a gift from Michael Gottesman; Addgene plasmid #10957; http://n2t.net/addgene:10957; RRID:Addgene_10957^127^) using Q5 High-Fidelity DNA Polymerase (New England Biolabs) and the following primers 5′-CACCTCGAGGATCTTGAAGGGGACCGCAATG; 3′-CACGCGGCCGCTCACTGGCGCTTTGTTCCAG. Product was cloned into a pcDNA5/FRT/TO-based vector modified to include an N-terminal Myc epitope tag^123^ and sequenced. This was then co-transfected into RKO Flp-In T-Rex cells, with the Flp-recombinase-encoding plasmid pOG44 (ThermoFisher Scientific), using Lipofectamine Plus (ThermoFisher Scientific) as described.^134^ Hygromycin (400 μg/mL; Roche)/Blasticidin (8 μg/mL; Melford)-resistant colonies were pooled, and transgene expression induced with 1 μg/mL tetracycline (Sigma-Aldrich).

#### Generation GFP-H2B expressing cells

OCMs and RKO cells expressing GFP-H2B were generated by lentiviral transduction as previously described.^37^ AAV293T cells (Agilent Technologies) were transfected with pLVX-myc-EmGFP-H2B^128^ along with psPAX2 and pMD2.G (gifts from Didier Trono; Addgene plasmid #12260; http://n2t.net/addgene:12260; RRID: Addgene_12260 and Addgene plasmid #12259; http://n2t.net/addgene:12259; RRID: Addgene_12259) using the ProFection Mammalian Transfection System (Promega). Virus was harvested 48 h later, centrifuged and filtered (0.45 μm), before being added with 10 μg/mL polybrene (Sigma-Aldrich) to OCMs or RKO cells that had been plated 24 h earlier into collagen-coated 12-well plates. Cells were then centrifuged at 300xg 30°C for 2.5 h. One milliliter of media was added and the plates incubated overnight. Puromycin selection (1 μg/mL; Sigma-Aldrich) was added 48 h post-transduction.

#### Time-lapse microscopy and analysis

For high-resolution mitotic analysis, cells were cultured on Primaria 24-well plates and drugs added 24 h later. Cells were then imaged every 10 min for 5 days, using an inverted microscope (Axiovert 200; Carl Zeiss, Inc) equipped with an automated Nano-DriveC stage (Mad City Labs, Inc.), and an environmental control chamber (Solent Scientific) to maintain cells at 37°C in a humidified stream of 5% CO_2_. A 32x LD A-Plan objective was used for imaging. Shutters, filter wheels and point visiting were driven by MetaMorph software (MDS Analytical Technologies) and images captured using a Evolve Delta camera (Photometics). Image sequences were exported and analyzed manually. Time spent in mitosis was measured from nuclear envelope break-down (NEBD) to anaphase onset from at least two biological replicates. Mitotic phenotypes were quantitated as *normal* if they successfully divided into two equal sized nuclei. Abnormal mitoses were defined as having either an anaphase *bridge*; undergoing anaphase with *unaligned* chromosomes; a *multipolar* mitosis; undergoing cohesion *fatigue;* or if the cell exited mitosis without dividing, mitotic *slippage*.

For drug-sensitivity and cell-fate profiling, cells expressing EmGFP-H2B were cultured on collagen-coated μclear 96-well plates (Greiner Bio-One). Inhibitors were added 24 h later and cells imaged using an IncuCyte S3 (Sartorius). Nine fields of view per well were captured either every 4 h for drug-sensitivity experiments or every 10 min for cell fate profiling. For drug sensitivity, IncuCyte S3 software was used in real-time to measure green-fluorescent object count. The Area Under the Curve (AUC) at each drug concentration was plotted against drug concentration to generate dose-response curves from which GI_50_ values were calculated. For cell fate profiling, phase images were exported in MPEG-4 and analyzed manually.^17^ Time spent in mitosis was measured from cell rounding up to division into daughter cells.

To measure apoptosis induction, tetracycline-inducible RKO/FRT/TO/Myc-Bcl-xL cells were plated into μclear 96-well plates (Greiner Bio-One) in the presence or absence of 1 μg/mL tetracycline (Sigma-Aldrich). 24 h later, a Bcl-xLi titration (A-1155463; 0, 1, 10, 25, 50, 100, 500 nM; Selleckchem), in the presence or absence of either 100 nM taxol (Sigma-Aldrich) or 1 μg/mL tetracycline, was performed in FluoroBrite DMEM-containing IncuCyte Caspase-3/7 Dye for Apoptosis Reagent (Sartorius). Cells were imaged (phase contrast and green fluorescence) every 2 h for 72 h using an IncuCyte S3 (Sartorius). IncuCyte software was used in real-time to measure confluency and green-fluorescence object count as an indicator of apoptosis. Data were imported into Excel (Microsoft) and Prism 10 (GraphPad) for analysis.

#### Cell biology

Immunofluorescence was performed as described previously,^37^^,^^93^^,^^135^ with the OCMs cultured on collagen-coated 19 mm coverslips. For p53 immunostaining OCMs were pretreated overnight with 10 μM Nutlin-3 (Sigma-Aldrich). For Mad2 staining OCMs were pretreated for 1 h with 0.2 μg/mL nocodazole (Sigma-Aldrich) with or without 2 μM Mps1i (AZ3146; Selleckchem), followed by the addition of 20 μM MG132 (Sigma-Aldrich) for another hour. Following fixation and permeabilization, the cells were incubated with the following primary antibodies for 30 min at room temperature: Sheep anti-Aurora A (1:1000)^123^; Sheep anti-Mad2 (1:500)^124^; Rabbit anti-Phospho-Histone H3 Ser10 (1:2000; Merck Millipore Cat#06–570, RRID:AB_310177); Rabbit anti-Pericentrin (1:1000; AbCam Cat#ab4448, RRID:AB_304461); Rabbit anti-MDR1 (1:100; Proteintech Cat#22336-1-AP, RRID:AB_2833023); human anti-ACA (1:500; kind gift from Prof. Bill Earnshaw^125^); Mouse anti-p53 (DO-1;1:1000; Santa Cruz Biotechnology; Cat#sc-126, RRID:AB_628082). Following washes, Cy2-and Cy3-conjugated secondary antibodies (1:500; Donkey anti-Sheep Cy3 Cat#713-165-147, RRID: AB_2315778; Donkey anti-Rabbit Cy2 Cat#711-225-152, RRID:AB_2340612; Donkey anti-Rabbit Cy3, cat#711-165-152, RRID:AB_2307443; Donkey anti-Human Cy2 Cat#709-225-149, RRID:AB_2340541; Donkey anti-Mouse Cy2: Cat#715-225-150, RRID:AB_2340826; Donkey anti-Mouse Cy3: Cat#715-165-150, RRID:AB_2340813; all Jackson ImmunoResearch Laboratories Inc) were added, before the DNA was counter stained with Hoechst 33358 (1 μg/mL; Sigma-Aldrich) and the coverslips mounted (90% glycerol. 20 mM Tris-HCl, pH 9.2) on to slides. Cells were analyzed and images taken using an Axioskop2 plus microscope (Carl Zeiss, Inc) and a CoolSNAP HQ camera (Photometrics). MetaMorph software (MDS Analytical Technologies) and Adobe Photoshop CC 2024 (Adobe Systems Inc.) were used to process images.

Immunoblotting was carried out as previously described.^131^ Cells were lysed in sample buffer, boiled for 5 min, then proteins resolved by SDS-PAGE, electroblotted onto Immobilon-P PVDF membranes (Merck Millipore), blocked in 5% skimmed milk (Marvel) and incubated overnight at 4°C with primary antibodies. When blotting for MDR1 cells pellets were lysed in sample buffer then incubated at 37°C for 10 min before SDS-PAGE. Mouse monoclonal anti-Myc-tag (4A6, 1:2000; Merck Millipore Cat#05–724, RRID:AB_309938); sheep anti-Bub3 (1:1000; A.J. Holland and S.S. Taylor, unpublished); Rabbit anti-MDR1 (1:1000; Proteintech Cat#22336-1-AP, RRID:AB_2833023); Rabbit anti-MDR1(D3H1Q;1:1000; Cell Signaling Technology Cat# 12683, RRID:AB_2715689). After washing, appropriate horseradish-peroxidase-conjugated secondary antibodies (Rabbit anti-Sheep IgG (HL) HRP Cat# G21040, RRID: AB_2536527 and Goat anti-Mouse IgG (HL) HRP Cat# G21234, RRID: AB_2536530, both Invitrogen, and Goat anti-Rabbit IgG (HL) HRP Merck Millipore Cat#ABC240, RRID: AB_2722647) were added for at least 2 h before visualisation using either EZ-Chemiluminescence Reagent (Geneflow Ltd) or Luminata Forte Western HRP Substrate (Merck Millipore) and a ChemiDoc Touch Imaging System (BioRad). Adobe Photoshop CC 2024 (Adobe Systems Inc.) was used to process images.

#### RNA sequencing of OCMs

RNA sequencing data from OCMs described previously are published (see resource availability).^37^^,^^120^^,^^121^^,^^122^ For RNA sequencing of OCMs first described here, total RNA extracted using a RNeasy Plus Mini kit (Qiagen) was submitted to the Genomic Technologies Core Facility. After quality and integrity assessment using a 4200 TapeStation (Agilent Technologies), libraries were generated using the Illumina Stranded mRNA Prep Ligation kit (Illumina, Inc.) according to the manufacturer’s protocol. Briefly, polyadenylated mRNA was purified from 0.025 to 1 μg total RNA using poly-T oligo-attached magnetic beads. mRNA was fragmented at elevated temperature before reverse transcription into first-strand cDNA using random hexamer primers in the presence of Actinomycin D. Following removal of template RNA, second-strand cDNA was synthesised to yield blunt-ended, double-stranded cDNA fragments (with strand specificity maintained by dUTP incorporation in place of dTTP to quench the second strand during subsequent amplification). Following a single adenine base addition, adapters with a complementary thymine overhang were ligated to the cDNA fragments followed by ligation of pre-index anchors to prepare for dual indexing. Index adapter sequences were added by PCR to enable multiplexing of the final cDNA libraries, which were pooled and loaded onto an SP flow cell and paired-end sequenced (59 + 59 cycles, plus indices) on an Illumina NovaSeq6000 instrument. Output data were demultiplexed and binary base call (BCL)-to-Fastq conversion performed using bcl2fastq software (Illumina, Inc., v2.20.0.422). Stranded paired-end reads were quality assessed using FastQC (v0.11.3)^136^ and FastQ Screen (v0.14.0),^137^ followed by adapter and low-quality base trimming with BBDuk from the BBMap suite (v36.32).^138^ Trimmed reads were mapped against the human reference genome (hg38) and gene annotation from Gencode (v32) using STAR (v2.7.2b).^129^ The “–quantMode GeneCounts” option was used to obtain read counts per gene from STAR. The R package DESeq2^139^ was then used to apply the median of ratios method of normalization. At the time of analysis, one OCM had not been interrogated by RNAseq, and four had *ABCB1* read counts of zero (Figures 6, S9A and S9B and Table S2). To ensure that data points corresponding to these OCMs appeared on Figure 6, where *ABCB1* expression is represented by bubble size, we allocated these subgroups the nominal small values of 0.1 and 0.5, respectively. In addition, complete historic taxol exposure data for six patients were not available, and 14 patients had not received taxol prior to sample collection (Figures S9A and S9B). Again, to facilitate data visualisation in Figure 6, these OCMs were allocated nominal small values of 10 and 50, respectively, such that ‘not known’ appears as white and ‘zero’ appears as black.

### Quantification and statistical analysis

Prism 10 (GraphPad) was used for statistical analysis, where ∗*p* < 0.05, ∗∗*p* < 0.01, ∗∗∗*p* < 0.001, ∗∗∗∗*p* < 0.0001, ns: *p* > 0.05. Details of statistical analyses are described in the figure legends.

## Supplemental information

Document S1. Figures S1–S10 and Table S1

Table S2. Normalized read counts for ABCB1 in OCMs indicated

Document S2. Article plus supplemental information
