# Supplementary material for: Screening a living biobank identifies cabazitaxel as a strategy to combat acquired taxol resistance in high-grade serous ovarian cancer
Source: Cell Rep Med. 2025 Jun 3;6(6):102160. doi: 10.1016/j.xcrm.2025.102160 (PMC12208324; doi:10.1016/j.xcrm.2025.102160)
Supplement: Document S1. Figures S1–S10 and Table S1 [file mmc1.pdf]

Cell Reports Medicine, Volume *6*

Supplemental information

Screening a living biobank identifies cabazitaxel as a strategy to combat acquired taxol resistance in high-grade serous ovarian cancer

Anthony Tighe, Louisa Nelson, Robert D. Morgan, Bethany M. Barnes, I-Hsuan Lin, Samantha Littler, James Altringham, Jean Ling Tan, Joanne C. McGrail, and Stephen S. Taylor

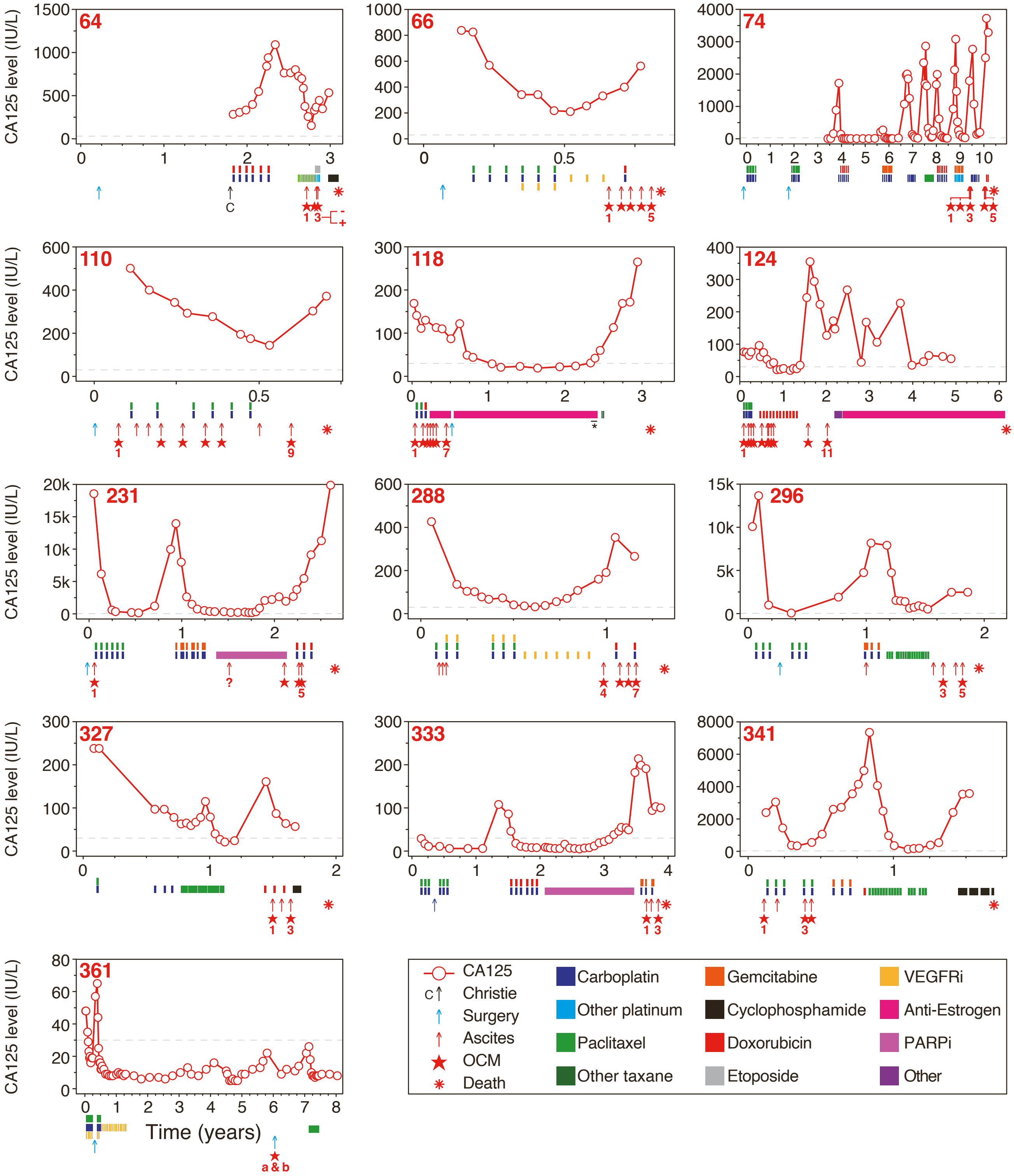
CA125 level (IU/L)
Time (years)
64
66
74
110
118
124
231
288
296
327
333
341
361
CA125
Christie
Surgery
Ascites
OCM
Death
Carboplatin
Other platinum
Paclitaxel
Other taxane
Gemcitabine
Cyclophosphamide
Doxorubicin
Etoposide
VEGFRi
Anti-Estrogen
PARPi
Other

Figure S1

Figure S1. Patient timelines for selected OCMs

Timelines for 13 patients, plotting CA125 values (proxy for disease progression) over time in years, annotated with the treatments received. Blue arrows show surgical sampling while red arrows show ascitic drains. Red stars indicate samples that gave rise to OCMs, and number/letter below indicating OCMs used in this study. ‘C’ indicates when the patient was referred to The Christie hospital; limited information is available prior to referral. Related to **Figure 1** and **Table S1**.

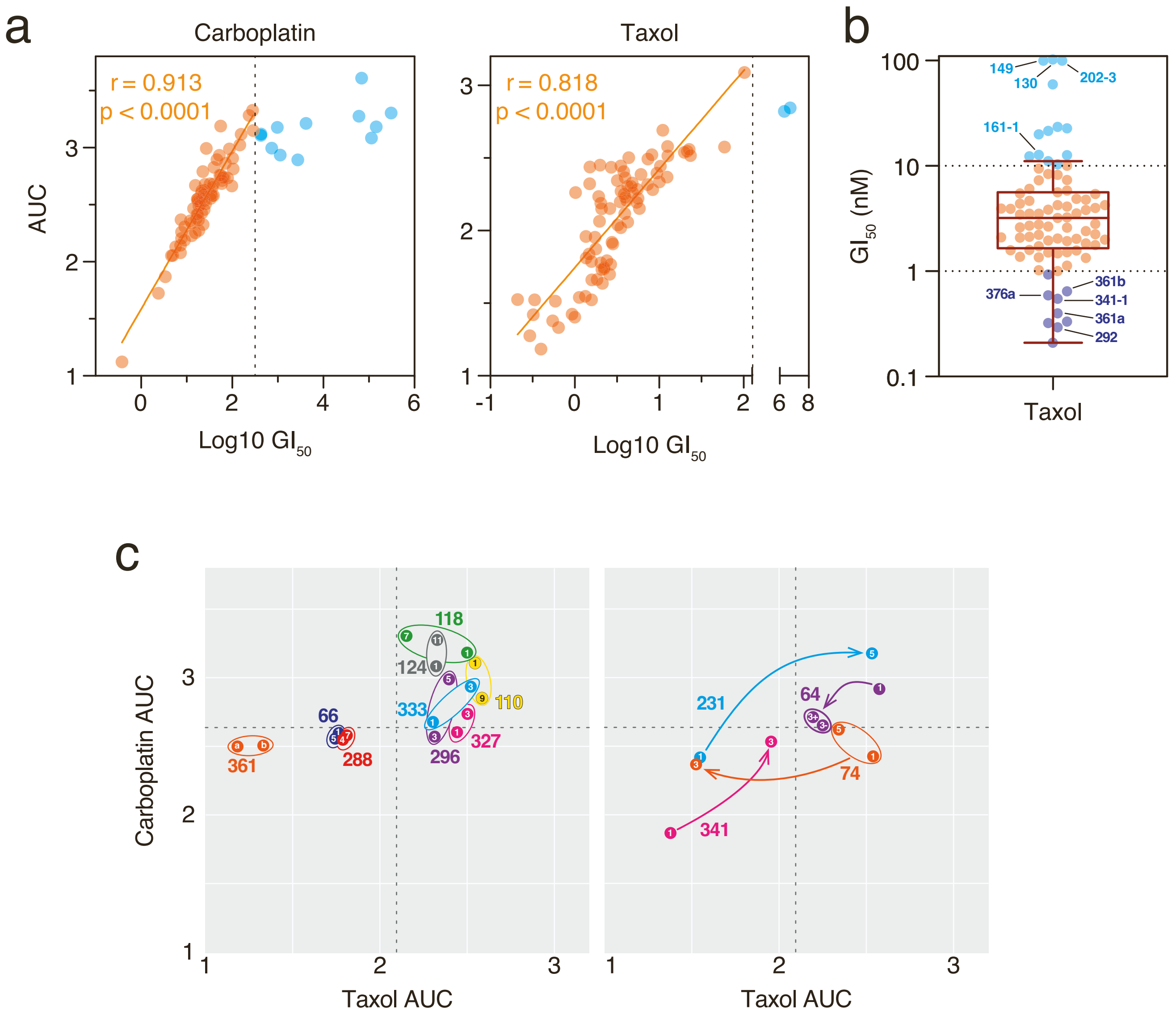
a
Carboplatin
r = 0.913
p < 0.0001
AUC
Log10 GI50
Taxol
r = 0.818
p < 0.0001
Log10 GI50
b
149
130
202-3
161-1
361b
376a
341-1
361a
292
GI50 (nM)
Taxol
c
Carboplatin AUC
Taxol AUC
118
124
110
333
66
296
327
361
288
231
64
74
341
Taxol AUC

Figure S2

Figure S2. OCM *ex vivo* drug sensitivity profiling captures inter-patient and intra-tumour heterogeneity

(**a**) *xy* graphs plotting area under the curve (AUC) versus GI_{50} values for carboplatin and taxol. OCMs with very high/indeterminant GI_{50} values are shown in cyan and excluded from correlation analysis. Orange lines show simple linear regressions, values represent Spearman r correlations (excluding 2 OCMs with indeterminate GI_{50} values). (**b**) Graph showing taxol GI_{50} values for each individual OCM, with the box-and-whiskers showing the interquartile ranges and Tukey fences. Highlighted in cyan are 13 OCMs with GI_{50} values greater than 10 nM, while nine OCMs with GI_{50} values less than 1 nM are highlighted in blue. (**c**) *xy* graph based on **Figure 1d**, plotting carboplatin AUC versus taxol AUC, highlighting 13 OCM subsets. See text for details. Related to **Figure 1**.

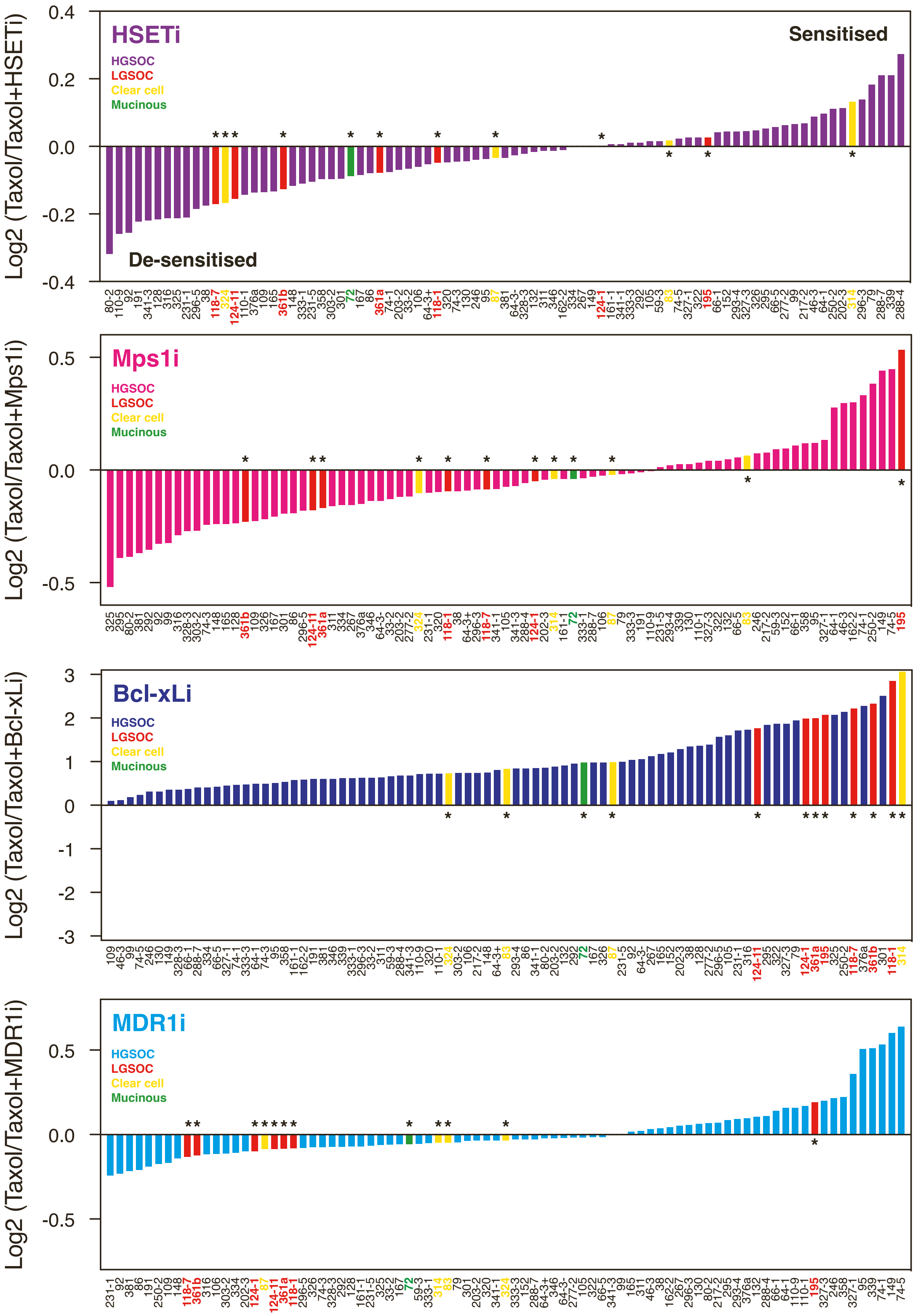
HSETi
HGSOC
LGSOC
Clear cell
Mucinous
Sensitised
De-sensitised
Log2 (Taxol/Taxol+HSETi)
Mps1i
HGSOC
LGSOC
Clear cell
Mucinous
Log2 (Taxol/Taxol+Mps1i)
Bcl-xLi
HGSOC
LGSOC
Clear cell
Mucinous
Log2 (Taxol/Taxol+Bcl-xLi)
MDR1i
HGSOC
LGSOC
Clear cell
Mucinous
Log2 (Taxol/Taxol+MDR1i)

Figure S3

Figure S3. Global analysis of taxol modulation strategies

Bar chart for each inhibitor plotting the mean log2 transformed AUC ratios. The abundant colour in each graph signifies the drug used in combination to maintain consistency throughout the manuscript (HSETi, purple; Mps1i, magenta; Bcl-xLi, blue; and MDR1i, cyan). The bars in less abundant colours highlight the 12 non-HGSOC OCMs which are also highlighted with an asterisk (LGSOC, red; Clear cell, yellow; Mucinous, green). Related to **Figure 2**.

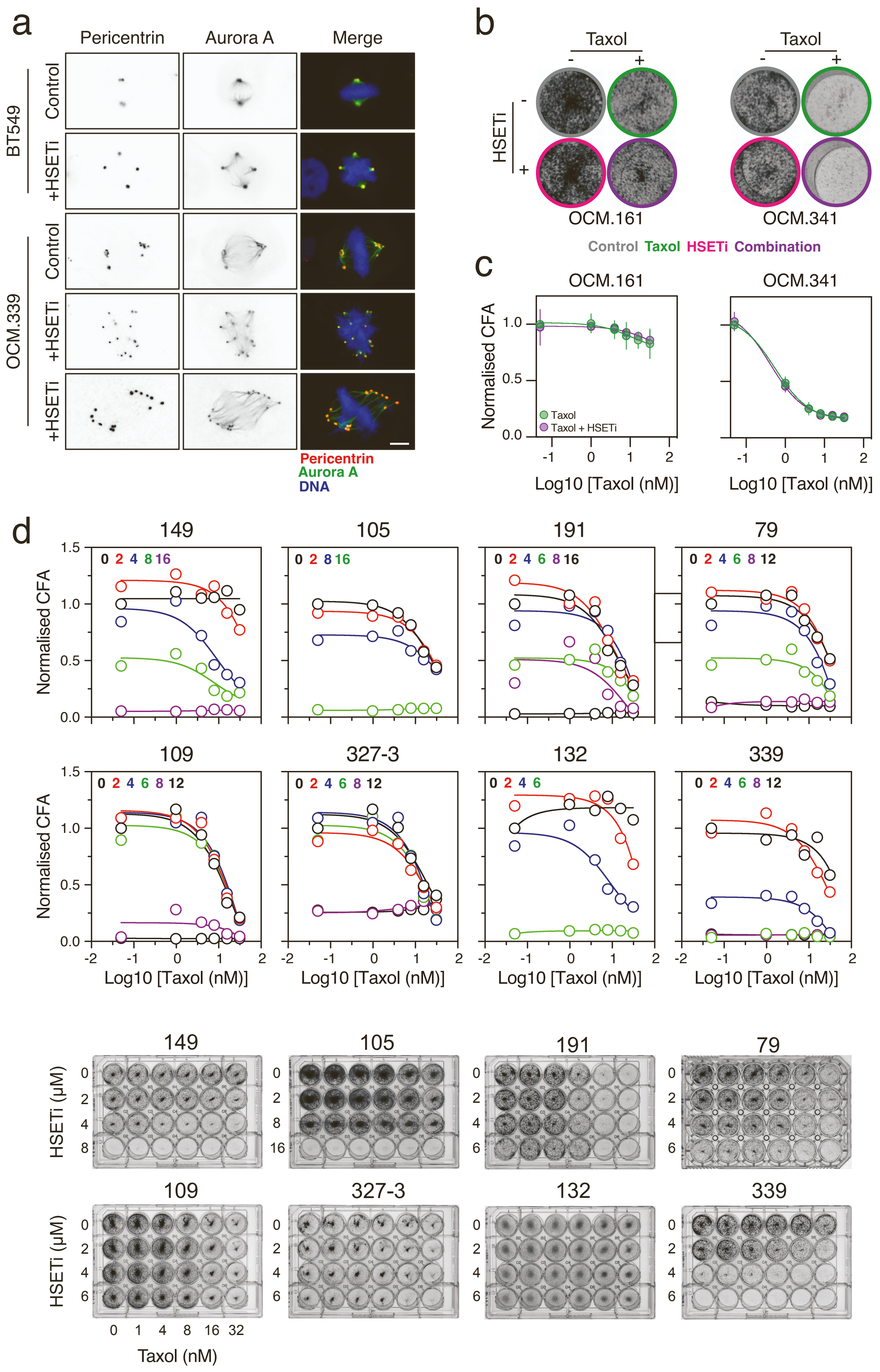
a
Pericentrin
Aurora A
Merge
BT549
Control
+HSETi
OCM.339
Control
+HSETi
+HSETi
Pericentrin
Aurora A
DNA
b
Taxol
HSETi
OCM.161
OCM.341
Control Taxol HSETi Combination
c
OCM.161
OCM.341
Normalised CFA
Taxol
Taxol + HSETi
Log10 [Taxol (nM)]
d
149
105
191
79
109
327-3
132
339
Normalised CFA
Log10 [Taxol (nM)]
HSETi (μM)
Taxol (nM)
0 1 4 8 16 32

Figure S4

Figure S4. Additional analysis of HSET inhibitor

(**a**) Immunofluorescence images of BT549 cells and OCM.339 treated ±2 μM HSETi for 24 h. Cells were fixed and stained to detect Pericentrin and Aurora A. Scale bar 10 μm. (**b**) Exemplar crystal violet-stained CFA of OCM.161 and OCM.341 following exposure to either a single dose of taxol (32 nM), HSETi (2 μM) or a combination of both. (**c**) Dose-response curves generated from CFA of OCM.161 and OCM.341 following exposure to either a taxol titration alone or in combination with 2 μM HSETi. Data are mean and SD from three biological replicates. (**d**) Top: Taxol dose-response curves generated from CFA for eight OCMs at HSETi concentrations up to 16 μM as indicated. Note that while higher HSETi concentrations are often toxic in the absence of taxol, in the case of OCM.149, 4 μM alone is relatively ineffective, but does cause a taxol sensitisation. These data are used in synergy analyses in **Figure S5b**. Bottom: Exemplar crystal violet-stained CFA used to generate dose-response curves. Related to **Figure 2**.

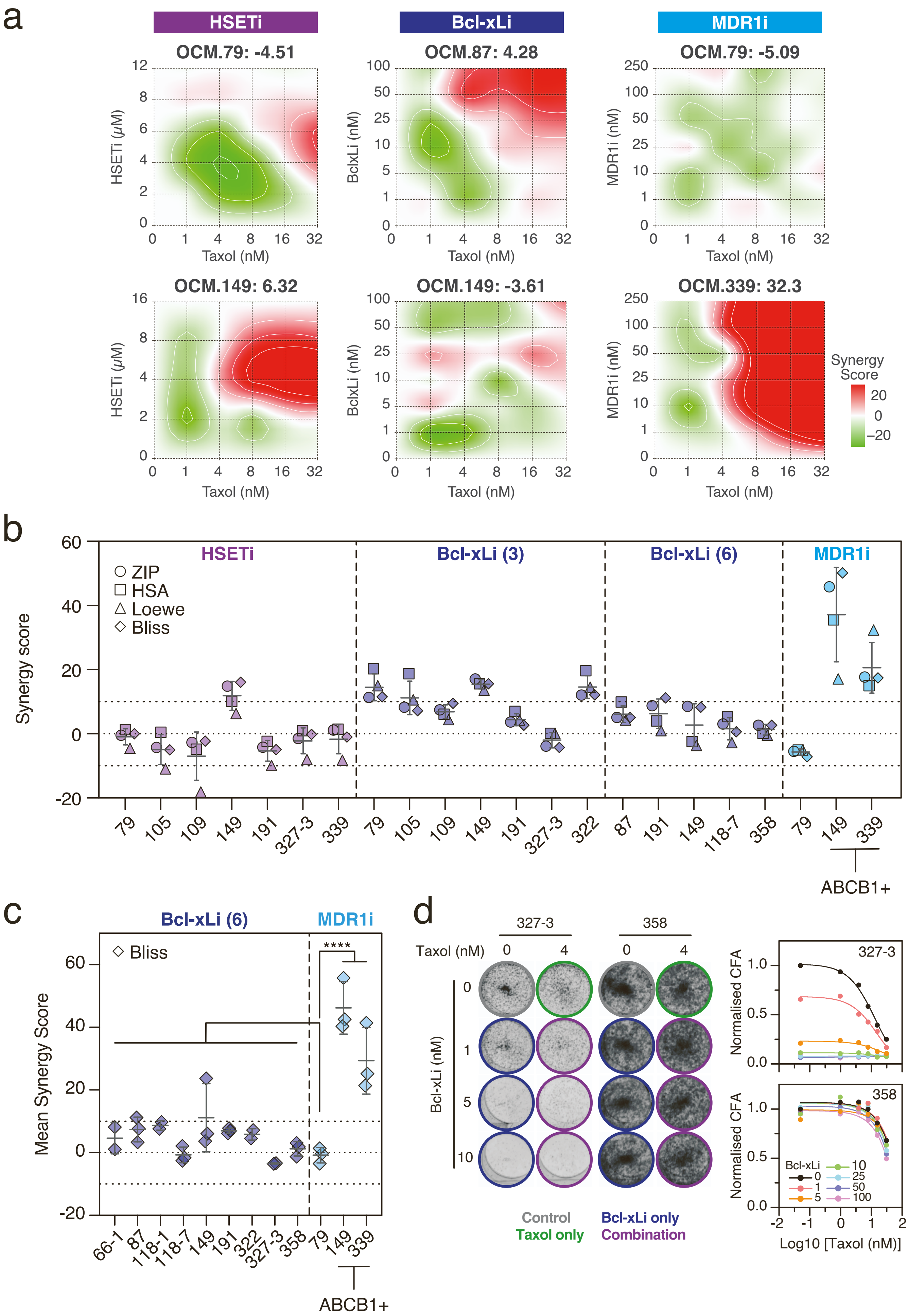
a
HSETi
Bcl-xLi
MDR1i
OCM.79: -4.51
OCM.87: 4.28
OCM.79: -5.09
OCM.149: 6.32
OCM.149: -3.61
OCM.339: 32.3
HSETi (μM)
BclxLi (nM)
MDR1i (nM)
Taxol (nM)
Synergy Score
b
Synergy score
HSETi
Bcl-xLi (3)
Bcl-xLi (6)
MDR1i
ZIP
HSA
Loewe
Bliss
ABCB1+
c
Bcl-xLi (6)
MDR1i
Bliss
Mean Synergy Score
****
ABCB1+
d
327-3
358
Taxol (nM)
Bcl-xLi (nM)
Control
Taxol only
Bcl-xLi only
Combination
Normalised CFA
Log10 [Taxol (nM)]
Bcl-xLi

Figure S5

Figure S5. Synergy analysis

(**a**) Exemplar synergy 2D heatmaps based on the Loewe model for the OCMs exposed to the drug combinations indicated. (**b**) Graph showing average synergy scores for the OCMs exposed to the taxol combinations indicated. The Bcl-xLi was tested using either a 3-point (10, 50, 100 nM) or 6-point titration (1, 5, 10, 25, 50, 100 nM). In each case, the average synergy score is shown for the four models deployed (ZIP (○), HSA (□), Loewe (△) and Bliss (◇)). Lines represent the average value of the four models plus SD. Dose-response curves are shown in **Figures S4** and **S7**. (**c**) Graph showing Bliss synergy scores for the OCMs exposed to the taxol combinations indicated. Each symbol represents independent biological replicates, with the bars showing the mean and SD. Statistical comparisons are with OCM.79 exposed to a taxol/MDR1i matrix. This OCM does not overexpress *ABCB1* so serves as a no-synergy control. OCMs 149 and 339 overexpress *ABCB1* resulting in statistically significant MDR1i synergy. One-way ANOVA, n.s. P>0.05, ****P<0.0001. In (**c**) and (**b**) the horizontal dashed lines mark synergy scores of -10, 0 and +10. Values greater than 10 suggest that the interaction between two drugs is likely to be synergistic. (**d**) Exemplar CFA for OCMs 327-3 and 358 and dose-response-curves. Related to **Figures 2, 4** and **5**.

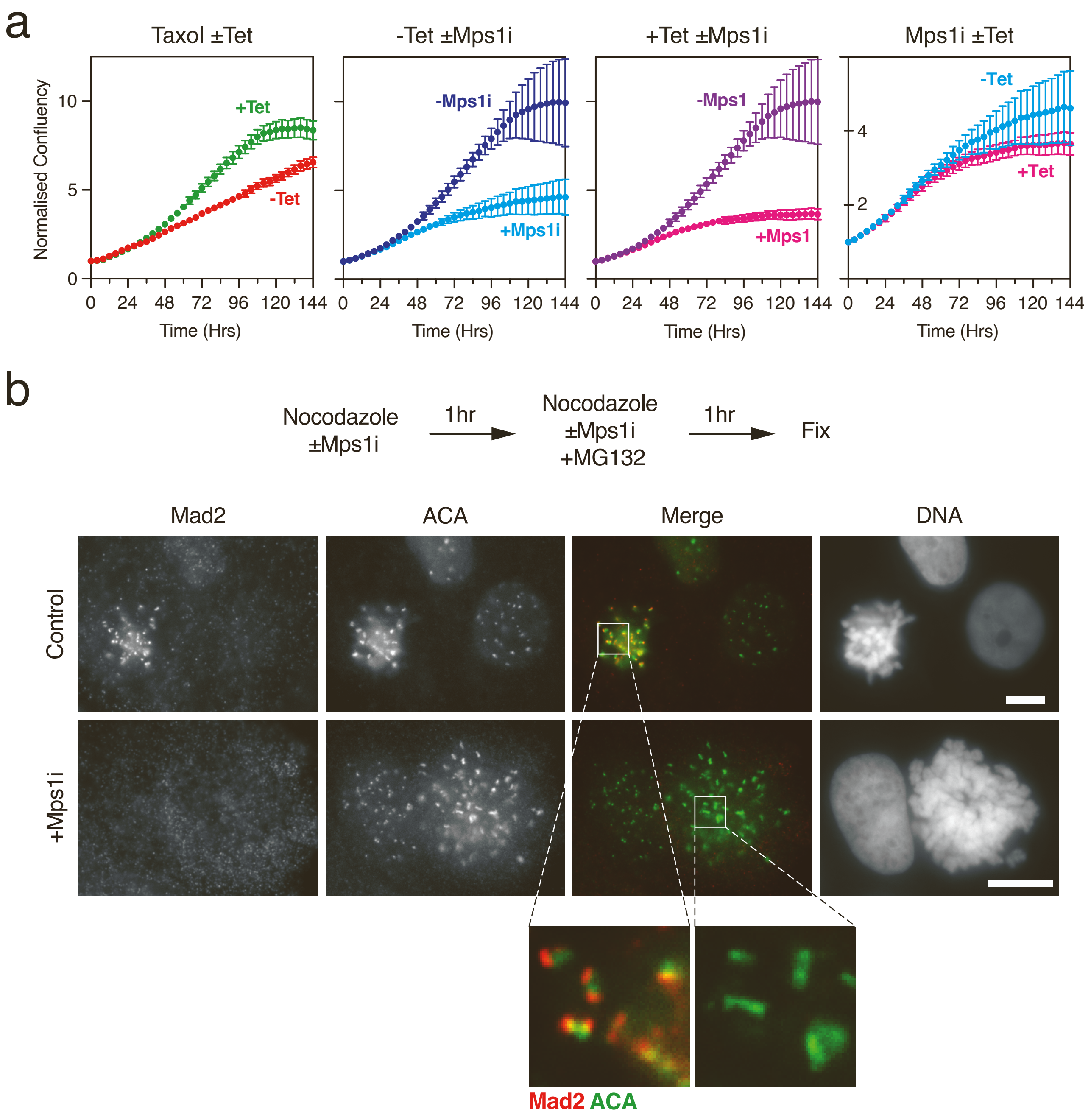
a
Taxol ±Tet
-Tet ±Mps1i
+Tet ±Mps1i
Mps1i ±Tet
Normalised Confluency
+Tet
-Tet
-Mps1i
+Mps1i
-Mps1
+Mps1
Time (Hrs)
b
Nocodazole ±Mps1i
1hr
Nocodazole ±Mps1i +MG132
Fix
Mad2
ACA
Merge
DNA
Control
+Mps1i
Mad2 ACA

Figure S6

Figure S6. Mps1i is not an MDR1 substrate

(**a**) IncuCyte dose-response curves for RKO GFP-H2B cells expressing Tet-inducible MDR1 ±2 μM Mps1i ±1 μg/ml Tet or 10 nM taxol ± 1 μg/ml Tet. Cells were imaged every 4 h for 6 days. Curves show the mean ± SD from three experiments. Note that while tet-induction of MDR1 attenuates the taxol effect, it has little effect on the Mps1i effect. Rather, tet-mediated MDR1 induction has a slight anti-proliferative effect, possibly due to increased ATP consumption (see right-hand graph). (**b**) Images of OCM.339 treated nocodazole ±Mps1i for 1 h, followed by nocodazole, MG132 ± Mps1i for 1 h. Cells were then fixed and stained to detect O-Mad2 and centromeres (ACA). Insets show higher magnification views of individual kinetochore pairs, showing Mad2 loss in the presence of the Mps1i. Scale bar, 10 μm. Related to **Figure 3**.

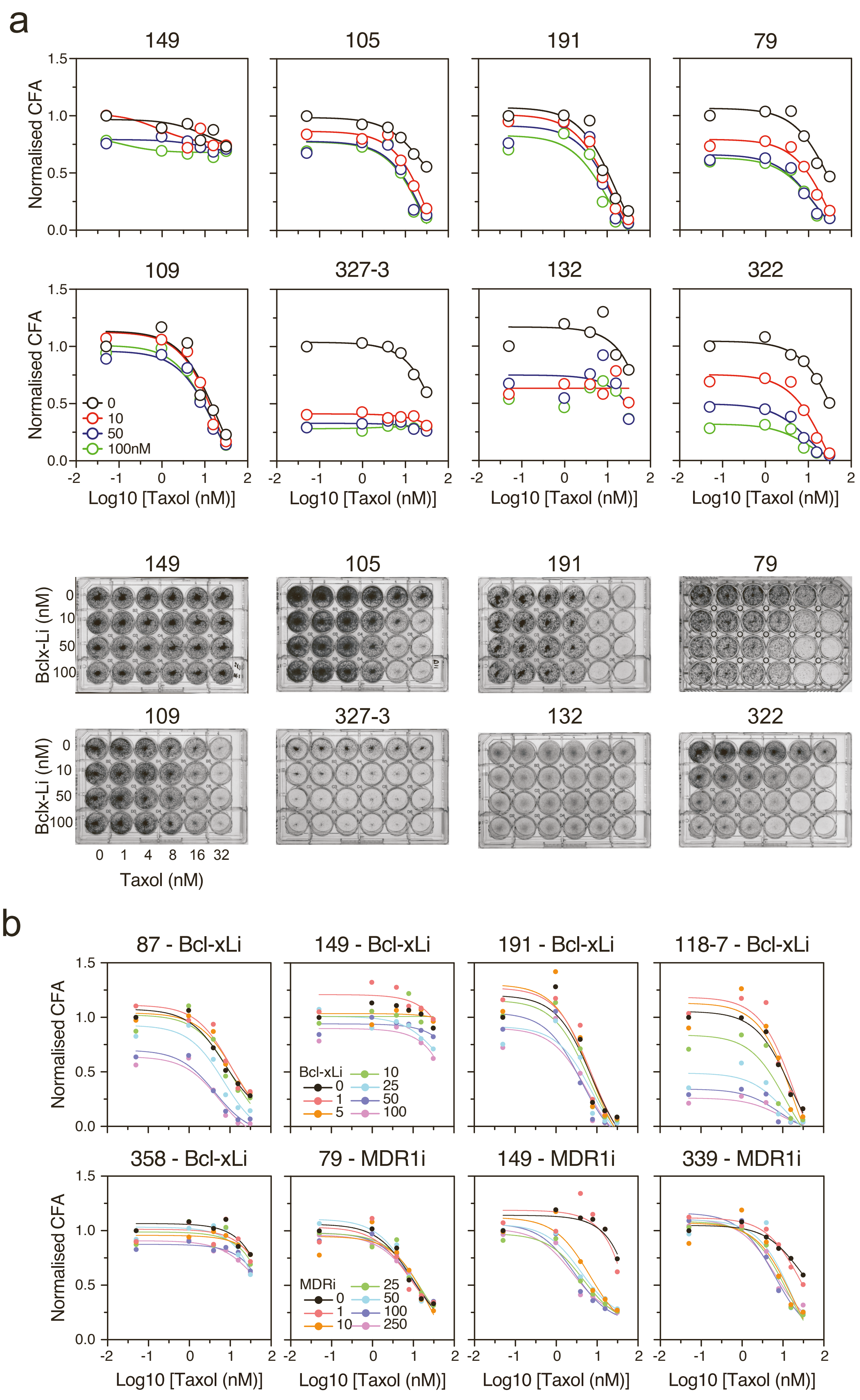
a
149
105
191
79
109
327-3
132
322
Normalised CFA
1.5
1.0
0.5
0.0
-2
-1
0
1
2
Log10 [Taxol (nM)]
0
10
50
100nM
Bclx-Li (nM)
0
10
50
100
0 1 4 8 16 32
Taxol (nM)
b
87 - Bcl-xLi
149 - Bcl-xLi
191 - Bcl-xLi
118-7 - Bcl-xLi
358 - Bcl-xLi
79 - MDR1i
149 - MDR1i
339 - MDR1i
Bcl-xLi
0
1
5
10
25
50
100
MDRi
0
1
10
25
50
100
250

Figure S7

Figure S7. Additional analysis Bcl-xL inhibitor

(**a**) Top: Taxol dose-response curves for eight OCMs at three Bcl-xLi concentrations (10, 50 and 100 nM) and images of associated colony formation assay plates. Bottom: Exemplar crystal violet-stained CFA used to generate dose-response curves. (**b**) Taxol dose-response curves for five OCMs at six Bcl-xLi concentrations (1, 5, 10, 25, 50 and 100 nM) and three OCMs at six MDR1i concentrations (1, 10, 25, 50, 100 and 250 nM). Data are used in synergy analyses in **Figure S5b**. Related to **Figures 4** and **5**.

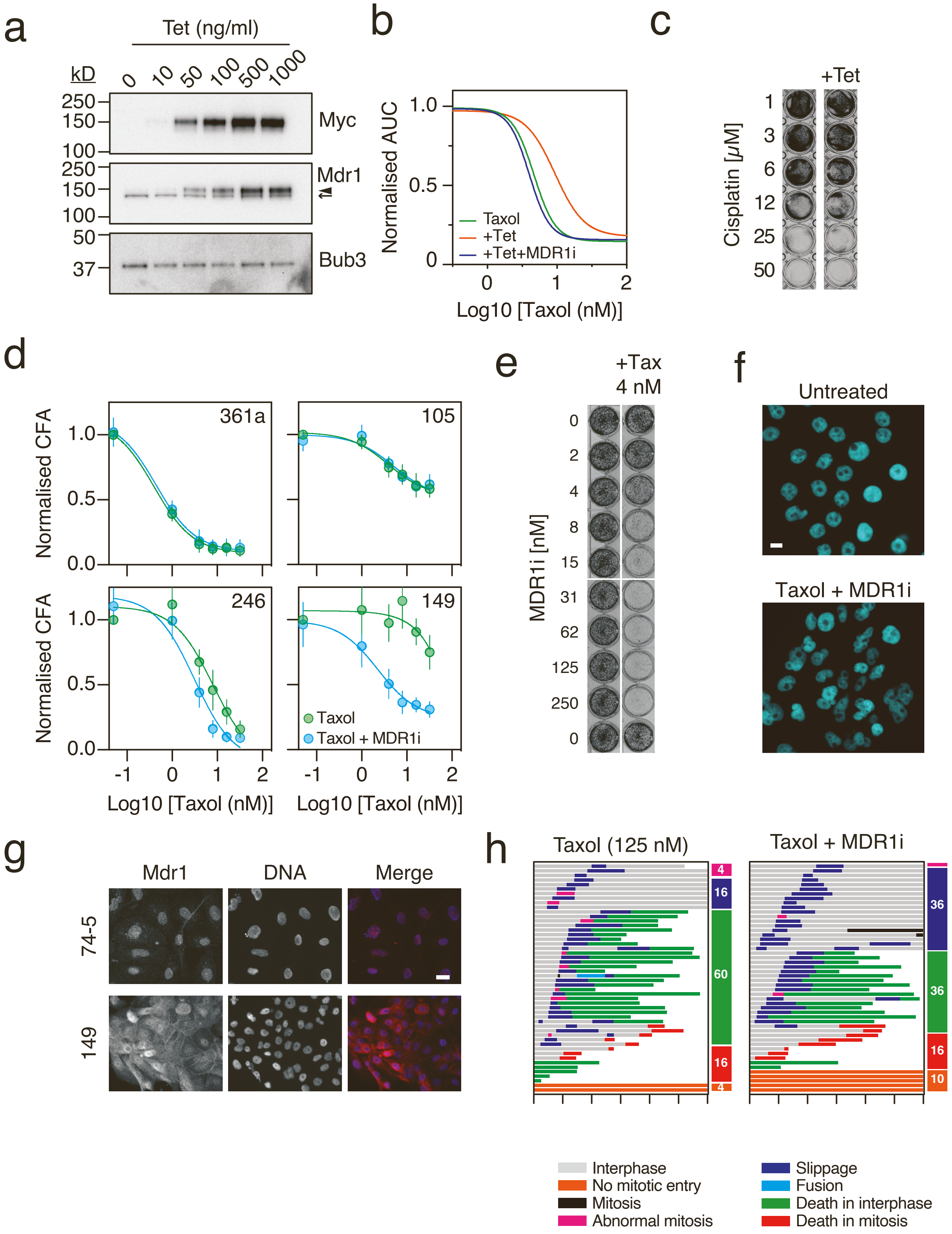
a
Tet (ng/ml)
kD
0
10
50
100
500
1000
250
150
100
Myc
Mdr1
50
37
Bub3
b
Normalised AUC
1.0
0.5
0
Taxol
+Tet
+Tet+MDR1i
Log10 [Taxol (nM)]
c
+Tet
Cisplatin [μM]
1
3
6
12
25
50
d
Normalised CFA
361a
105
246
149
Taxol
Taxol + MDR1i
Log10 [Taxol (nM)]
e
+Tax
4 nM
MDR1i [nM]
0
2
4
8
15
31
62
125
250
f
Untreated
Taxol + MDR1i
g
Mdr1
DNA
Merge
74-5
149
h
Taxol (125 nM)
Taxol + MDR1i
Interphase
No mitotic entry
Mitosis
Abnormal mitosis
Slippage
Fusion
Death in interphase
Death in mitosis

Figure S8

Figure S8. Inhibition of MDR1i-mediated drug efflux re-sensitises a subset of OCMs to taxol

(**a**) Immunoblot of RKO cells expressing a tetracycline (Tet)-inducible, Myc-tagged MDR1. Arrow points to endogenous and arrowhead points to ectopic Mdr1. Bub3 is used as a loading control. (**b**) IncuCyte® dose-response curves for RKO GFP-H2B cells expressing Tet-inducible MDR1 in the presence of a taxol titration ±1 μg/ml Tet and 250 nM MDR1i. (**c**) Exemplar CFA of RKO cells expressing Tet-inducible MDR1, exposed to a titration of cisplatin ±1 ug/ml Tet. Cells were imaged every 6 h for 5 days. Curves show the average from three biological replicates. (**d**) Dose-response curves for OCMs highlighted in **Figure 5b**. Data are mean and SD from three biological replicates. (**e**) CFA images of OCM.149 treated with a MDR1i titration ±4 nM taxol. (**f**) DNA-stained nuclei of OCM.149. Cells exposed to 4 nM taxol and MDR1i show nuclear atypia. Scale bar 10 μm. (**g**) Immunofluorescence images of OCMs 74-5 and 149 showing expression of MDR1. Scale bar 20 μm. (**h**) Cell fate profiling of OCM.149 treated with either taxol (125 nM) or with taxol (125 nM) + MDR1i for 6 days. Horizontal bars represent a single cell (50 cells per condition), with colours indicating cell behaviour. Numbers in coloured boxes show the percentage of cells with the indicated behaviour. Related to **Figure 5**.

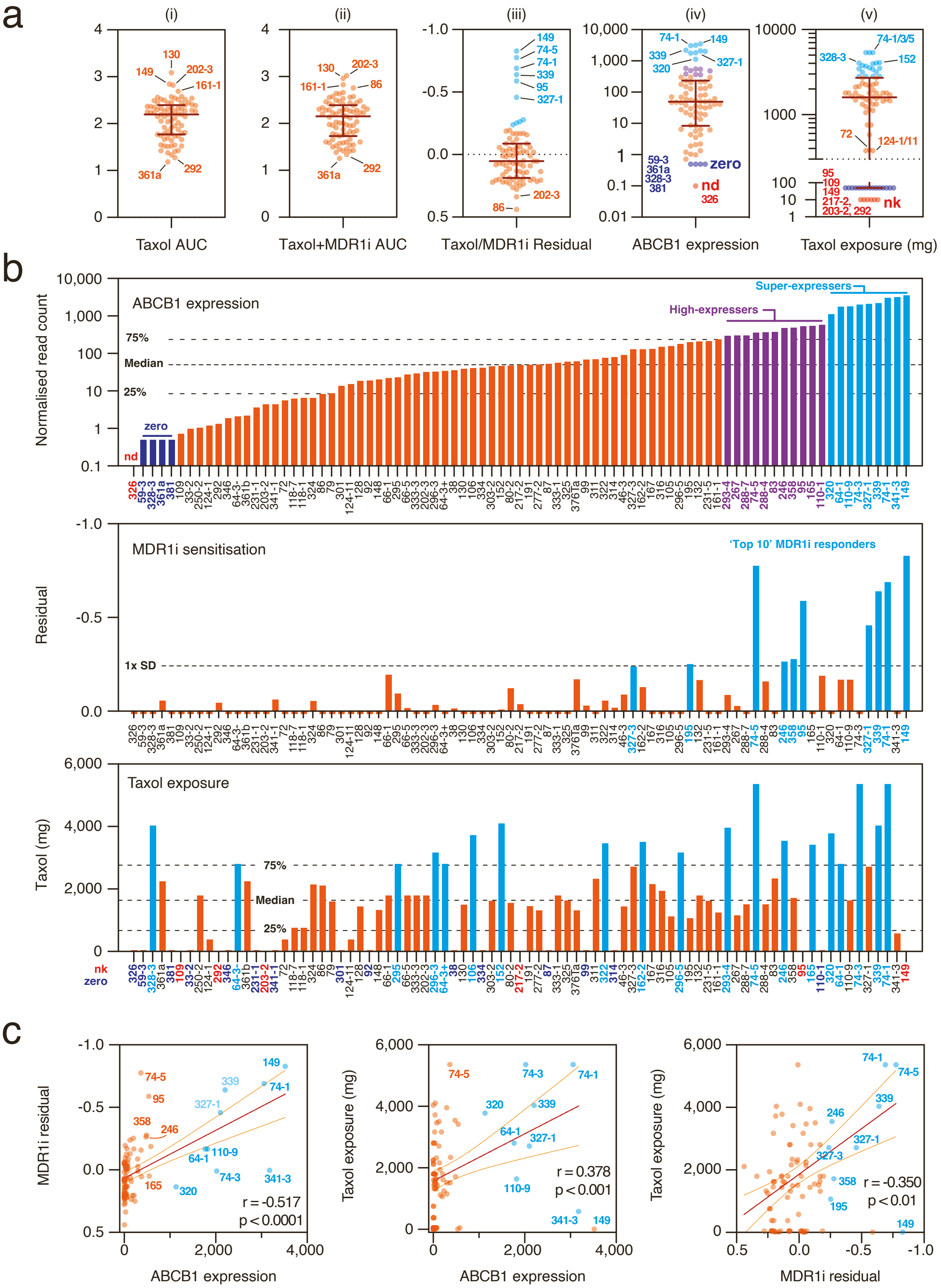
a
(i)
(ii)
(iii)
(iv)
(v)
Taxol AUC
Taxol+MDR1i AUC
Taxol/MDR1i Residual
ABCB1 expression
Taxol exposure (mg)
b
ABCB1 expression
Normalised read count
Super-expressers
High-expressers
75%
Median
25%
zero
nd
MDR1i sensitisation
Residual
'Top 10' MDR1i responders
1x SD
Taxol exposure
Taxol (mg)
nk
zero
c
MDR1i residual
ABCB1 expression
r = -0.517
p < 0.0001
Taxol exposure (mg)
r = 0.378
p < 0.001
MDR1i residual
r = -0.350
p < 0.01

Figure S9

Figure S9. Acquired taxol resistance correlates with *ABCB1* overexpression

(**a**) Dot plots for 83 OCMs showing: response to either (i) taxol alone (Taxol AUC) or (ii) in combination with MDR1i (Taxol+MDR1i AUC); (iii) Extent of re-sensitisation to taxol by MDR1i (Taxol/MDR1i Residual); (iv) *ABCB1* expression levels determined by OCM RNAseq (normalised read count; four OCMs had zero expression (dark blue); one OCM had no data available = nd (red)); (v) Milligrams of taxol each patient received prior to sample collection (14 dark blue OCMs received zero taxol prior to sample collection; for six OCMs taxol exposure was not known = nk). (**b**) Bar charts showing: (*top*) *ABCB1* expression levels determined by OCM RNAseq (normalised read count), highlighting "*high-expressers*" and "*super-expressers*"; (*middle*) Top 10 MDR1i-responders in the screen, note only negative residuals shown to identify MDR1i responders; (*bottom*) Amount of taxol each patient received prior to sample collection. (**c**) Pairwise *xy* graphs of: (i) MDR1i residual against *ABCB1* expression; (ii) Patient taxol exposure (mg) against OCM *ABCB1* expression; (iii) Patient taxol exposure (mg) against OCM MDR1i residual. Spearman r is used to measure the correlation. Highlighted in blue are *ABCB1* super-expressers (left and middle) or top MDR1i responders (right). Note that in the centre and right graphs, for the six OCMs where the taxol exposure is 'not known', the data point is eliminated from the correlation calculation. Related to **Figure 6** and **Table S2**.

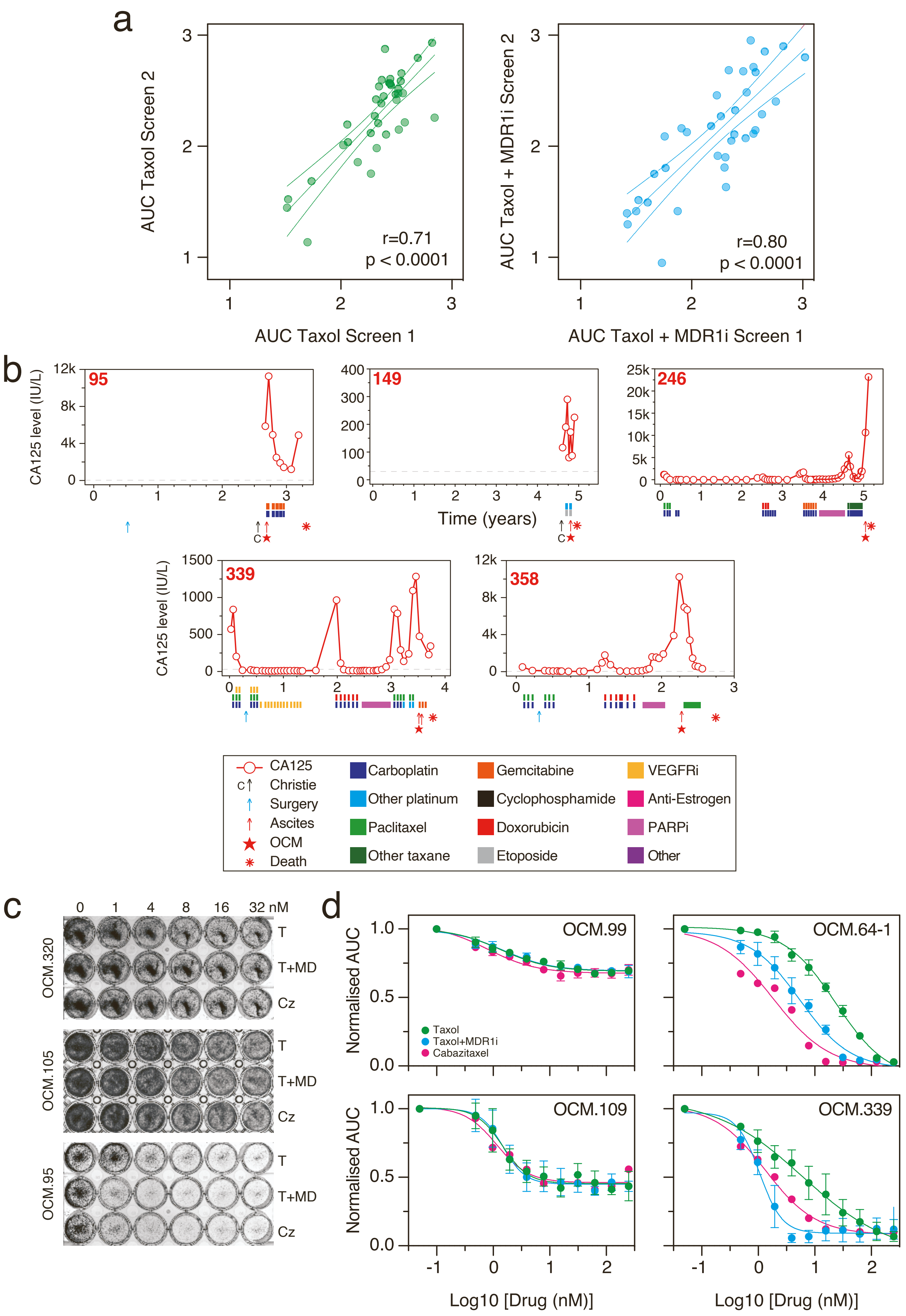

a
AUC Taxol Screen 2
AUC Taxol Screen 1
r=0.71
p < 0.0001
AUC Taxol + MDR1i Screen 2
AUC Taxol + MDR1i Screen 1
r=0.80
p < 0.0001
b
CA125 level (IU/L)
95
149
246
339
358
Time (years)
CA125
Christie
Surgery
Ascites
OCM
Death
Carboplatin
Other platinum
Paclitaxel
Other taxane
Gemcitabine
Cyclophosphamide
Doxorubicin
Etoposide
VEGFRi
Anti-Estrogen
PARPi
Other
c
0 1 4 8 16 32 nM
OCM.320
OCM.105
OCM.95
T
T+MD
Cz
d
Normalised AUC
OCM.99
OCM.64-1
OCM.109
OCM.339
Taxol
Taxol+MDR1i
Cabazitaxel
Log10 [Drug (nM)]

Figure S10

Figure S10. OCMs re-sensitised to taxol via MDR1i are sensitive to cabazitaxel monotherapy

(**a**) *xy* graphs showing the correlation between the response of 36 OCMs to taxol alone (left panel) or to taxol plus MDR1i (right panel) in the first screen compared with the second screen. Spearman r is used to measure the correlation. (**b**) Timelines for five patients, plotting CA125 values (proxy for disease progression) over time in years, annotated with the treatments received. Blue arrows show surgical sampling while red arrows show ascitic drains. Red stars indicate samples that gave rise to OCMs, and number/letter below indicating OCMs used in this study. 'C' indicates when the patient was referred to The Christie hospital; limited information is available prior to referral. (**c**) Exemplar CFA images of OCMs 95, 105 and 320 fixed and stained after exposure to either a taxol titration ±MDR1i (MD), or a cabazitaxel (Cz) titration. (**d**) IncuCyte® dose-response curves for GFP-H2B-expressing OCMs 99, 64-1, 109 and 339 in the presence of either a taxol titration ±MDR1i, or a cabazitaxel titration. Cells were imaged every 2 or 4 h for a minimum of 120 h. Graphs show mean and SEM from three biological replicates. Related to **Figure 7**.

Patient					OCM							
#	ID	FIGO Stage	Subtype[a]	*TP53* Primary tumour (targeted amplicon)	OCM ID[b]	Chemo -naïve (Y/N)[c]	Biopsy type	*TP53* (sequencing of cloned transcripts)		p53 immunostaining +/- Nutlin-3		References
								DNA	Protein prediction	-Nutlin-3[d]	+Nutlin-3[e]	
1	33	4B	HGSOC	NA	33-2	N	Ascites	c.783insATT	Inframe insertion	+	-	2, 3, 4, 5
2	38	3C	HGSOC	c.376-1G>C	38	Y	Ascites	c.375_395del	Inframe deletion	-	-	1, 2, 3, 5
3	46	3C	HGSOC	c.267delC	46-3	N	Ascites	c.267delC	Frameshift	-	-	2, 3, 4, 5
4	59	3C	HGSOC	c.398delT	59-3	N	Ascites	c.398delT	Frameshift	-	-	2, 3, 4, 5
5	64	3C	Possible mixed LGSOC/HGSOC[†]	c.646G>A	64-1	N	Ascites	c.646G>A	p.V216M	+	-	2, 3, 4, 5
					64-3-Ep-	N	Ascites	c.646G>A	p.V216M	+	-	2, 3, 5
					64-3-Ep+			c.646G>A	p.V216M	+	-	2, 3
6	66	3C	HGSOC	Neoplastic cell count less than 10%	66-1	N	Ascites	c.488A>G	p.Y163C	+	-	2, 3, 4, 5
					66-5	N	Ascites	c.488A>G	p.Y163C	+	-	2, 3, 5
7	72	1A	Moderately differentiated MOC	c.843C>G	72	N	Ascites	c.843C>G	p.D281E	+	-	2, 3, 5
8	74	3B	HGSOC	c.1024C>T	74-1	N	Ascites	c.1024C>T	p.R342X	+	-	2, 3, 4
					74-3	N	Ascites	c.1024C>T	p.R342X	+	-	2, 3
					74-5	N	Ascites	c.1024C>T	p.R342X	ND	ND	This study
9	79	3C	HGSOC	NA	79	N	Ascites	c.153_162del	Frameshift	-	-	2, 3, 5
10	80	3C	HGSOC	c.742C>T	80-2	N	Ascites	c.742C>T	p.R248W	+	ND	3, 4
11	83	3A2	CCOC	ND	83	N	Ascites	WT	WT	-	+	This study
12	86	4B	HGSOC	NA	86	N	Ascites	c.842A>G	p.D281G	-	-	3, 4
13	87	3B	Possible CCOC[†]	NA	87	Y	Ascites	WT	WT	+	+	2, 3, 4, 5
14	92	3C	HGSOC	WT	92	N	Ascites	c.524G>A	p.R175H	-	ND	3, 4
15	95	3C	HGSOC	Insufficient DNA	95	N	Ascites	c.902del	Frameshift	+	-	4
16	99	3C	HGSOC	Insufficient DNA	99	Y	Ascites	c.810T>G	p.F270L	+	ND	3, 4
17	105	3C	HGSOC	Insufficient DNA	105	N	Ascites	c.431A>C	p.Q105P	+	-	4, 5
18	106	3C	HGSOC	NA	106	N	Ascites	c.844C>T	p.R282W	+	-	4
19	109	4B	HGSOC	Insufficient DNA	109	N	Ascites	c.743G>A	p.R248Q	+	-	3, 4, 5, 6
20	110	3C	HGSOC	ND	110-1	Y	Ascites	c.743G>A	p.R248Q	+	-	1, 3, 4
				ND	110-9	N	Ascites	ND	ND	ND	ND	3

21	118	3C	LGSOC	ND	118-1	Y	Ascites	ND	ND	-	+	1, 3
					118-7	N	Ascites	ND	ND	-	+	3
22	124	3C	LGSOC	ND	124-1	Y	Ascites	ND	ND	-	+	1, 3
					124-11	N	Ascites	c.524G>A	p.R175H	-	+	3
23	128	3C	HGSOC	NA	128	N	Ascites	c.514_559del	Frameshift	+	-	4
24	130	4B	HGSOC	ND	130	N	Ascites	c.536A>C	p.H179P	-	-	This study
25	132	3C	HGSOC	c.742C>T	132	N	Ascites	c.742C>T	p.R248W	+	ND	4
26	148	3C	HGSOC	ND	148	N	Ascites	c.713G>A	p.C238Y	+	-	7
27	149	3C	HGSOC	NA	149	N	Ascites	c.724T>G	p.C242G	+	-	3, 4
28	152	3C	Moderately differentiated serous adenocarcinoma of intermediate grade[†]	NA	152	N	Ascites	c.659A>G	p.Y220C	+	-	3, 4, 5
29	161	4A	HGSOC	NA	161-1	N	Ascites	c.524G>A	p.R175H	-	ND	3, 4
30	162	3C	HGSOC	NA	162-2	N	Ascites	Exon 7 deletion	-	-	ND	4
31	165	3C	HGSOC	c.659A>G	165	N	Ascites	c.659A>G	p.Y220C	+	ND	3, 4
32	167	3C	HGSOC	c.742C>T[f]	167	N	Ascites	c.742C>T	p.R248W	+	ND	3, 4
33	191	3A	HGSOC	c.743G>A	191	N	Ascites	c.743G>A	p.R248Q	+	ND	3, 4, 5
34	195	4A	Possible LGSOC[†]	WT	195	Y	Ascites	WT	WT	+	+	3, 4, 5
35	202	3C	HGSOC	ND	202-3	N	Ascites	c.818G>A	p.R273H	+	+	This study
36	203	3C	HGSOC	c.1025G>C	203-2	N	Ascites	c.1025G>C	p.R342P	+	ND	4
37	217	3C	HGSOC	ND	217-2	N	Solid	c.993_994 ins328	Frameshift	ND	ND	This study
38	231	3C	HGSOC	NA	231-1	Y	Ascites	c.742C>G	p.R248G	+	-	3, 4
					231-5	N	Ascites	c.742C>T	p.R248W	+	-	7
39	246	4A	HGSOC	c.309C>G	246	N	Ascites	c.309C>G	p.Y103*	-	ND	4, 6
40	250	3C	HGSOC	c.584T>A	250-2	N	Ascites	c.584T>A	p.I195N	+	+	4
41	267	4B	HGSOC	NA	267	N	Ascites	c.659A>G	p.Y220C	+	ND	4
42	277	4B	HGSOC	ND	277-2	N	Ascites	c.742C>T	p.R248W	+	-	This study
43	288	4B	HGSOC	ND	288-4	N	Ascites	c.743G>A	p.R248Q	+	-	This study
					288-7	N	Ascites	c.743G>A	p.R248Q	+	-	This study
44	292	3C	HGSOC	ND	292	N	Ascites	c.63delC	fs. Stop 43	-	-	This study
45	293	4B	HGSOC	ND	293-4	N	Ascites	c.659A>G	p.Y220C	ND	ND	This study
46	295	3C	HGSOC	ND	295	N	Ascites	c.757_758insA	Frameshift	-	-	This study
47	296	3C	HGSOC	ND	296-3	N	Ascites	c.542G>C	p.R181P	ND	ND	This study
					296-5	N	Ascites	c.542G>C	p.R181P	+	-	This study
48	301	4B	HGSOC	ND	301	Y	Ascites	c.524G>A	p.R175H	-	-	This study
49	303	4A	HGSOC	ND	303-2	N	Ascites	c.916C>T	p.R306*	-	-	This study

50	311	3C	OC	ND	311	N	Ascites	c.734G>A	p.G245D	+	-	This study
51	314	1A	CCOC	ND	314	Y	Ascites	WT	WT	-	+	This study
52	316	3C	HGSOC	ND	316	N	Ascites	c.371insT	fs. Stop 148	-	-	This study
53	320	3C	HGSOC	ND	320	N	Ascites	c.742C>T	p.R248W	+	-	This study
54	322	3A	HGSOC	ND	322	N	Ascites	c.400T>G	p.F134V	+	-	This study
55	324	3C	CCOC	ND	324	N	Ascites	C.524G>A	p.R175H	-	-	This study
56	325	4B	HGSOC	ND	325	N	Ascites	c.723delC	Frameshift	-	-	This study
57	326	4B	HGSOC	ND	326	Y	Ascites	ND	ND	ND	ND	This study
58	327	3C	HGSOC	ND	327-1	N	Ascites	c.523C>G	p.R175G	+	-	This study
					327-3	N	Ascites	c.523C>G	p.R175G	+	-	This study
59	328	3C	HGSOC	ND	328-3	N	Ascites	c.733G>A	p.G245S	ND	ND	This study
60	333	3C	HGSOC	ND	333-1	N	Ascites	c.524G>A	p.R175H	-	-	This study
					333-3	N	Ascites	c.524G>A	p.R175H	ND	ND	This study
61	334	3C	HGSOC	ND	334	Y	Ascites	c.743G>T	p.R248L	+	-	This study
62	339	4A	HGSOC	ND	339	N	Ascites	c.527G>A	p.C176Y	+	-	This study
63	341	3C	HGSOC	ND	341-1	Y	Ascites	c.503del26	-	-	-	This study
				ND	341-3	N	Ascites	c.524G>A	p.R175H	ND	ND	This study
64	346	3C	HGSOC	ND	346	N	Ascites	c.375_395del	p.T125_K132del	ND	ND	This study
65	358	3C	HGSOC	ND	358	N	Ascites	ND	ND	-	-	This study
66	361	3C	LGSOC	ND	361a	N	Solid	c.524G>A	p.R175H	-	-	This study
					361b	N	Solid	c.912delT	fs. Stop 344	ND	ND	This study
67	376	3C	HGSOC	ND	376a	N	Solid	c.818G>A	p.R273H	ND	ND	This study
68	381	3C	HGSOC	ND	381	Y	Ascites	c.376_396del	p.T125_K132del	ND	ND	This study

Table S1. Patient and OCM characteristics

a, Based on histology and other information where indicated by † (see Barnes *et al*. 2021).

b, The models are referred to using the OCM prefix followed by the patient number and, if one of a longitudinal series, the biopsy number. Models generated independently from the same biopsy are distinguished by an alphabetical suffix.

c, At the time of the research biopsy.

d, p53 status without Nutlin-3 treatment: '-' indicates p53 not detected, '+' indicates p53 detected.

e, p53 status following Nutlin-3 treatment: '-' indicates no induction of p53, '+' indicates induction of p53.

f, This *TP53* variant was detected at a variant allele frequency of 6% in a tumour block with >50% tumour cell content.

CCOC, clear cell ovarian cancer; fs, frameshift; HGSOC, high-grade serous ovarian cancer; LGSOC, low-grade serous ovarian cancer; MOC, mucinous ovarian cancer; NA, not available; ND, not done; OC, ovarian cancer; WT, wildtype.

References: 1) Pillay, N., *et al*. (2019) DNA Replication Vulnerabilities Render Ovarian Cancer Cells Sensitive to Poly(ADP-Ribose) Glycohydrolase Inhibitors. Cancer Cell, 35, 519-533 e518. 2) Nelson, L., Tighe, A., *et al*. (2020) A living biobank of ovarian cancer *ex vivo* models reveals profound mitotic heterogeneity. Nat Commun, 11, 822. 3) Barnes, B.M., *et al*. (2021) Distinct transcriptional programs stratify ovarian cancer cell lines into the five major histological subtypes. Genome Med, 13, 140. 4) Coulson-Gilmer, C., *et al*. (2021) Replication catastrophe is responsible for intrinsic PAR glycohydrolase inhibitor-sensitivity in patient-derived ovarian cancer models. J Exp Clin Cancer Res, 40, 323. 5) Golder, A., *et al*. (2022) Multiple-low-dose therapy: effective killing of high-grade serous ovarian cancer cells with ATR and CHK1 inhibitors. NAR Cancer, 4, zcac036. 6) Coulson-Gilmer, C., *et al*. (2024) Intrinsic PARG inhibitor sensitivity is mimicked by *TIMELESS* haploinsufficiency and rescued by nucleoside supplementation. NAR Cancer, 6. zcae030. 7) Littler, S., *et al.* (2025) Targeting SUMOylation in ovarian cancer: sensitivity, resistance, and the role of MYC. iScience. 112555.
